# Supplementary figures and images for: WhyD tailors surface polymers to prevent premature bacteriolysis and direct cell elongation in Streptococcus pneumoniae
Source: eLife. 2022 May 20;11:e76392. doi: 10.7554/eLife.76392 (PMC9208761; doi:10.7554/eLife.76392)

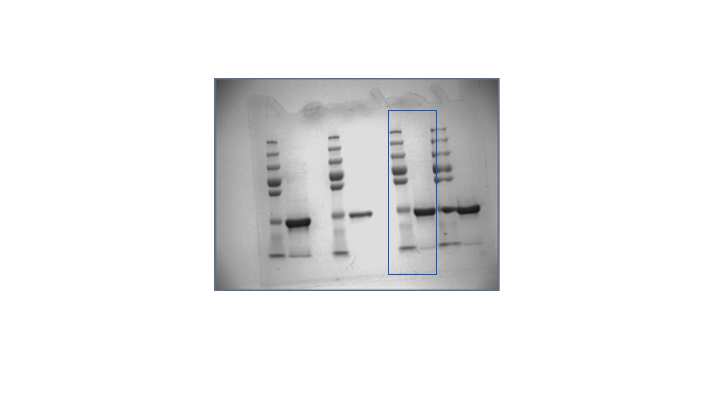

Supplement: Figure 1—source data 1. [file elife-76392-fig1-data1.zip › Figure 1 - source data/Figure 1D - source data /Figure 1D - source data_LytA_protein gel_labeled.tiff]

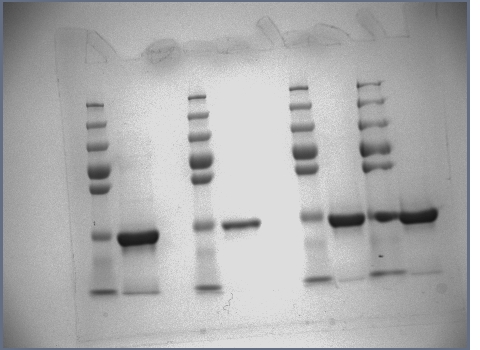

Supplement: Figure 1—source data 1. [file elife-76392-fig1-data1.zip › Figure 1 - source data/Figure 1D - source data /Figure 1D - source data_LytA_protein gel.tiff]

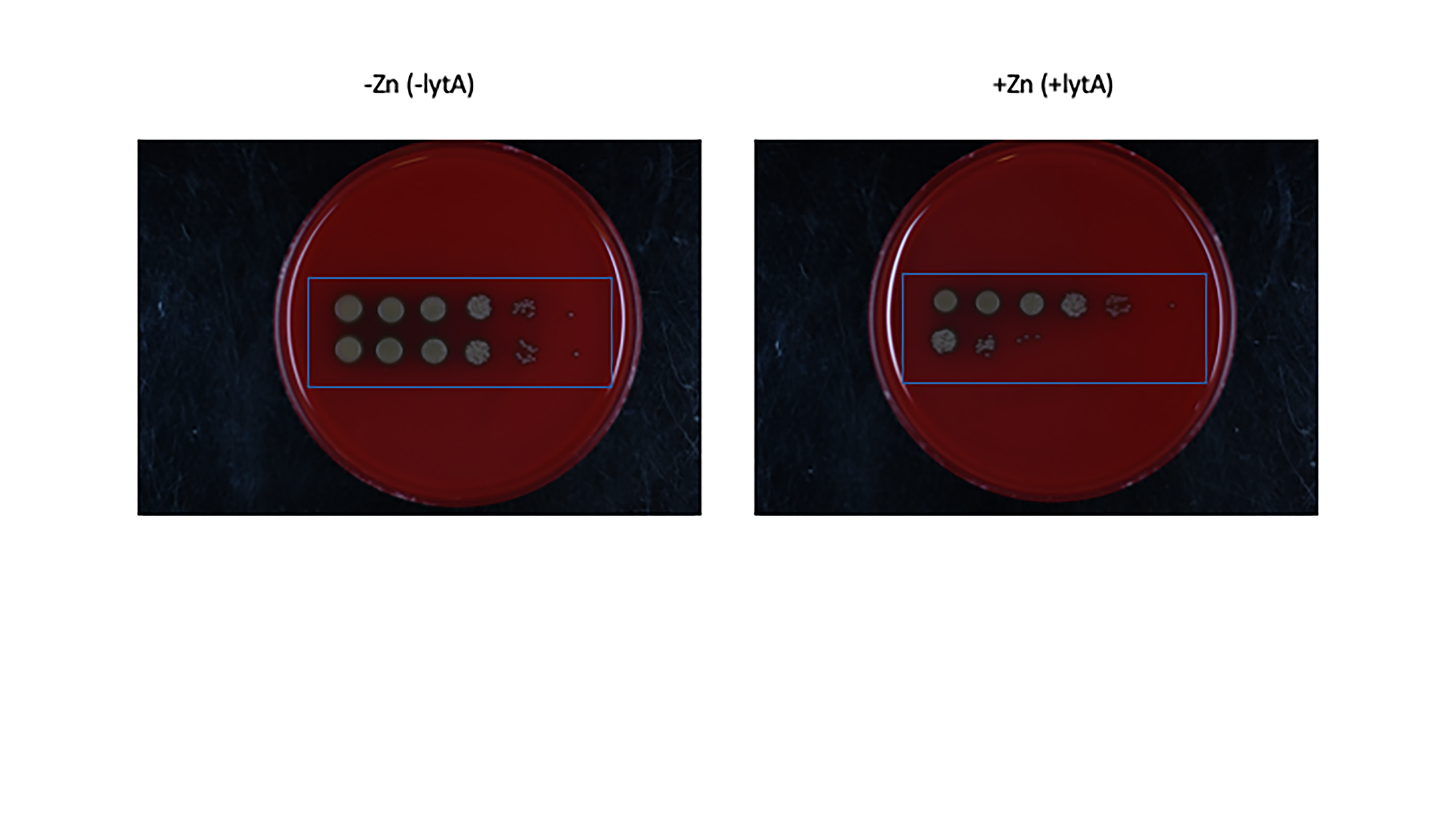

Supplement: Figure 1—source data 1. [file elife-76392-fig1-data1.zip › Figure 1 - source data/Figure 1B - source data /Figure 1B- source data_labeled.tiff]

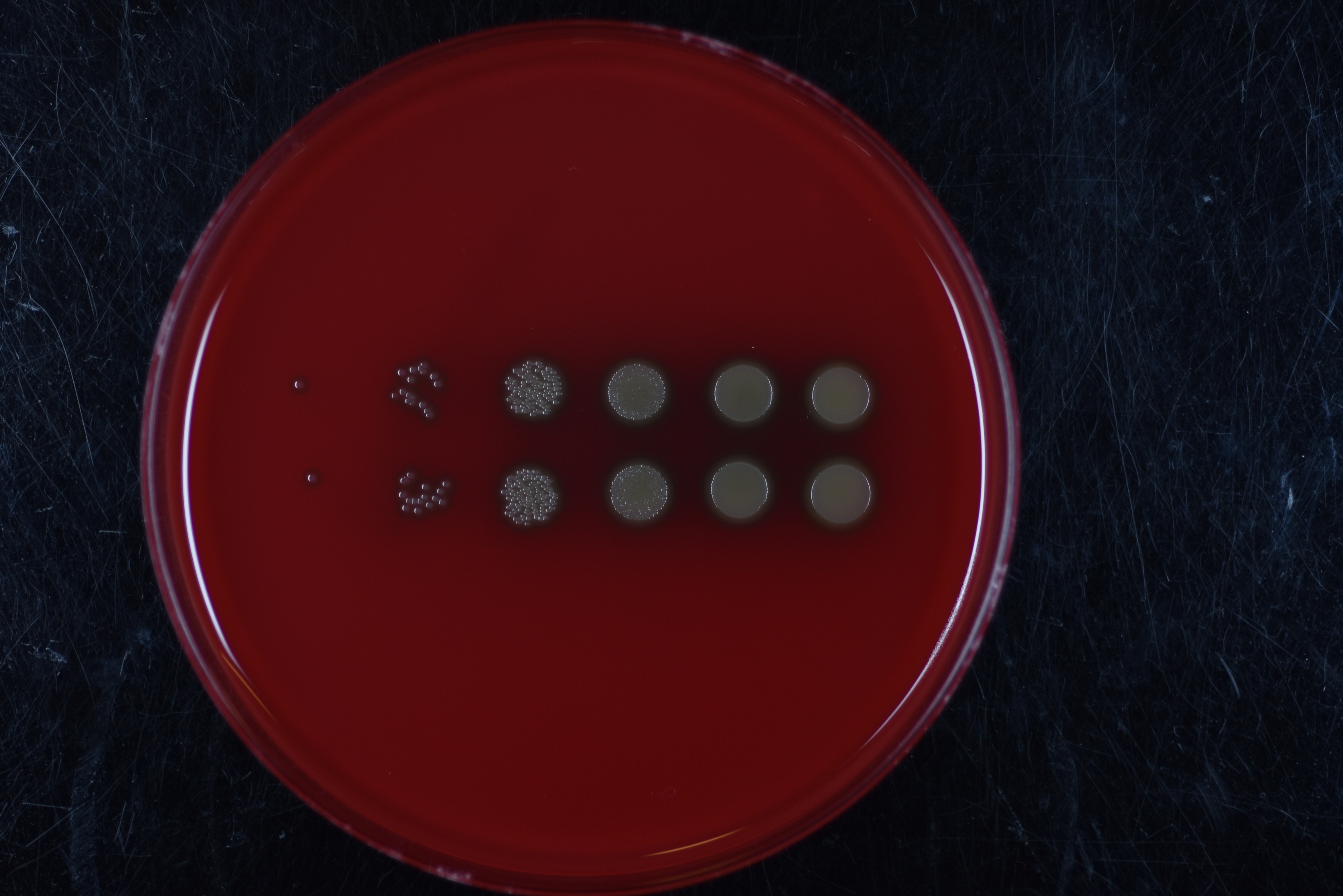

Supplement: Figure 1—source data 1. [file elife-76392-fig1-data1.zip › Figure 1 - source data/Figure 1B - source data /Figure 1B - source data_-Zn (-lytA).tiff]

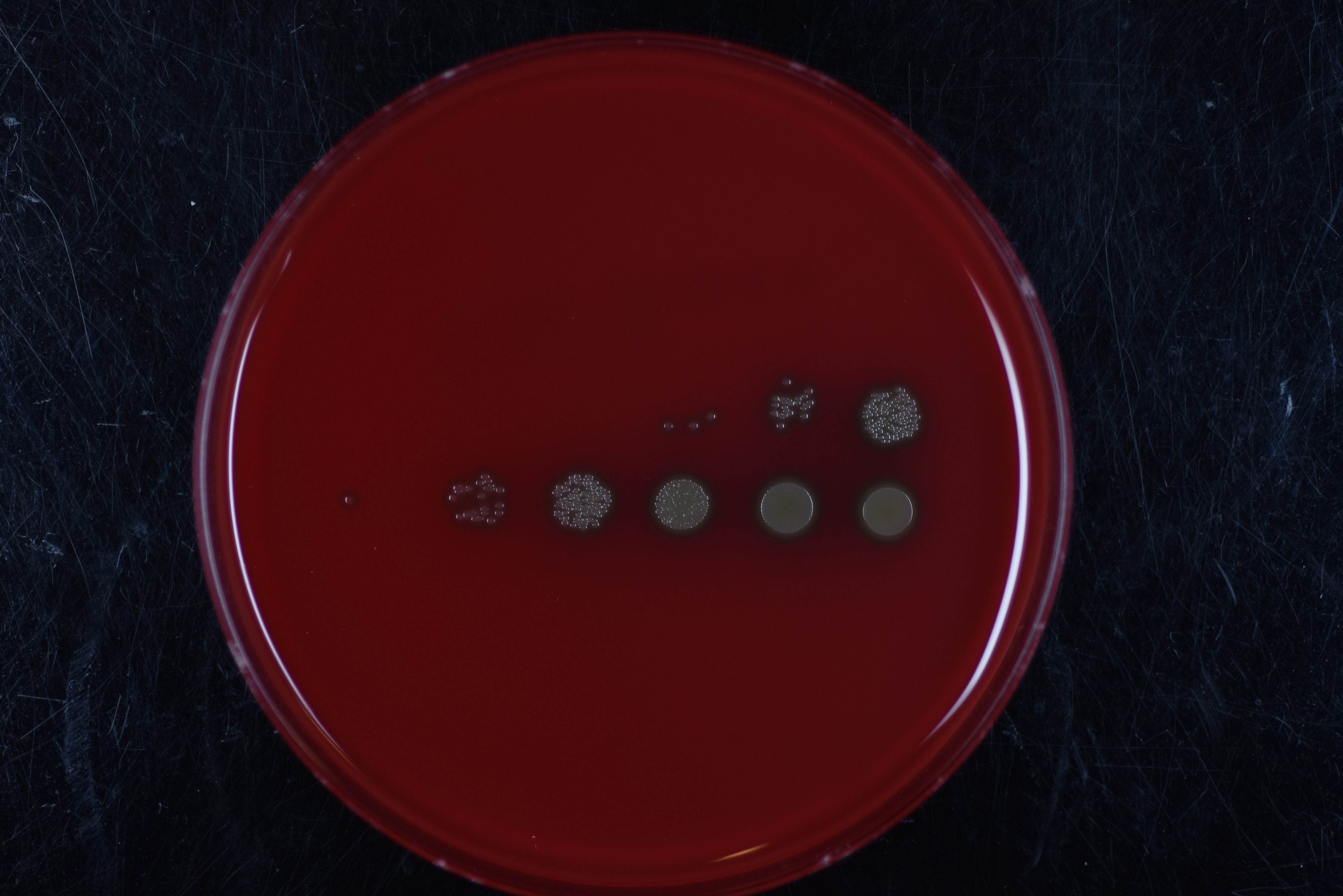

Supplement: Figure 1—source data 1. [file elife-76392-fig1-data1.zip › Figure 1 - source data/Figure 1B - source data /Figure 1B - source data_+Zn (+lytA).tiff]

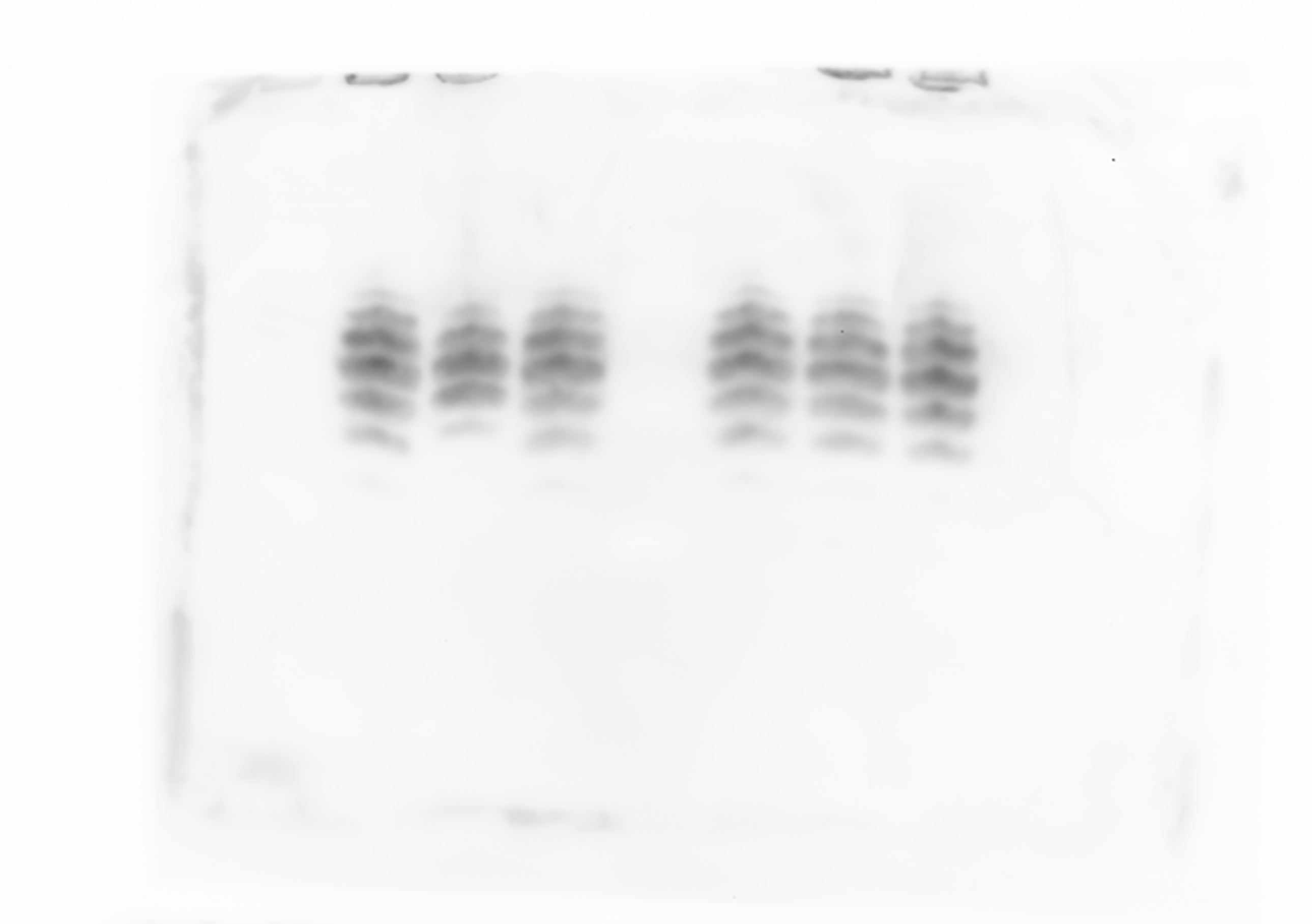

Supplement: Figure 2—source data 1. [file elife-76392-fig2-data1.zip › Figure 2 - source data/Figure 2 - source data/Figure 2- source data_Bottom panel_LTA.tif]

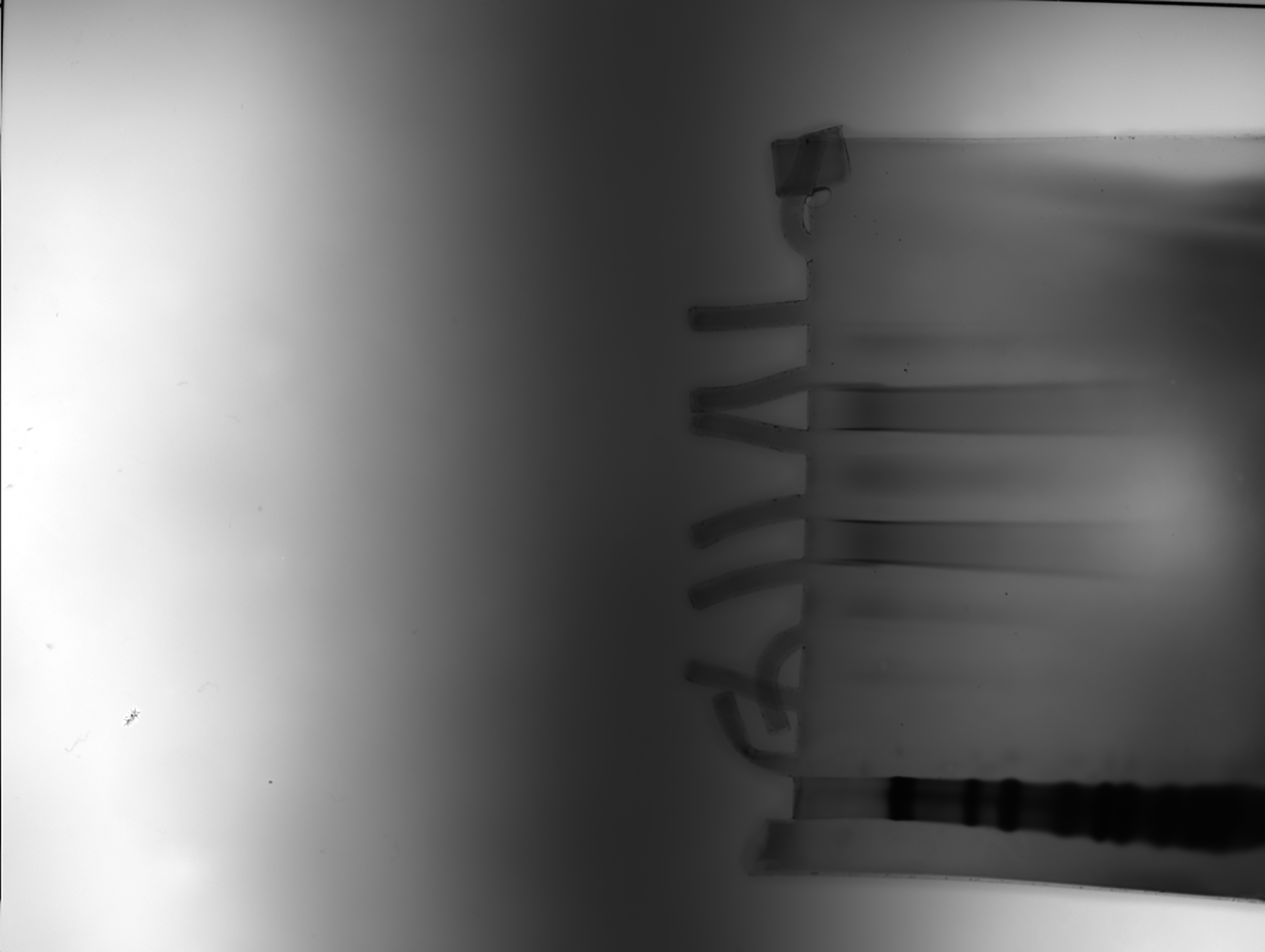

Supplement: Figure 2—source data 1. [file elife-76392-fig2-data1.zip › Figure 2 - source data/Figure 2 - source data/Figure 2- source data_Top panel_WTA.tiff]

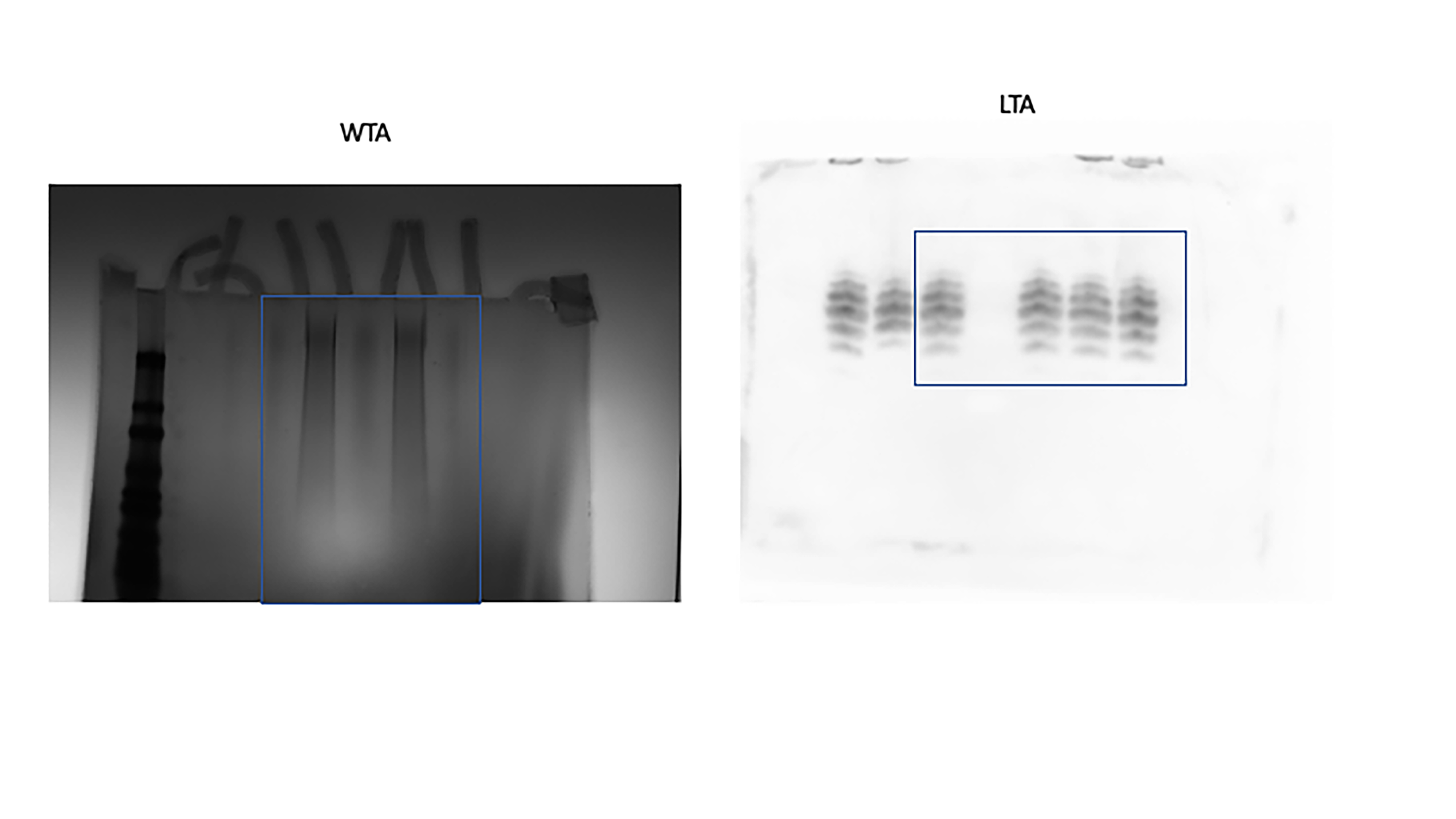

Supplement: Figure 2—source data 1. [file elife-76392-fig2-data1.zip › Figure 2 - source data/Figure 2 - source data/Figure 2- source data_labeled.tiff]

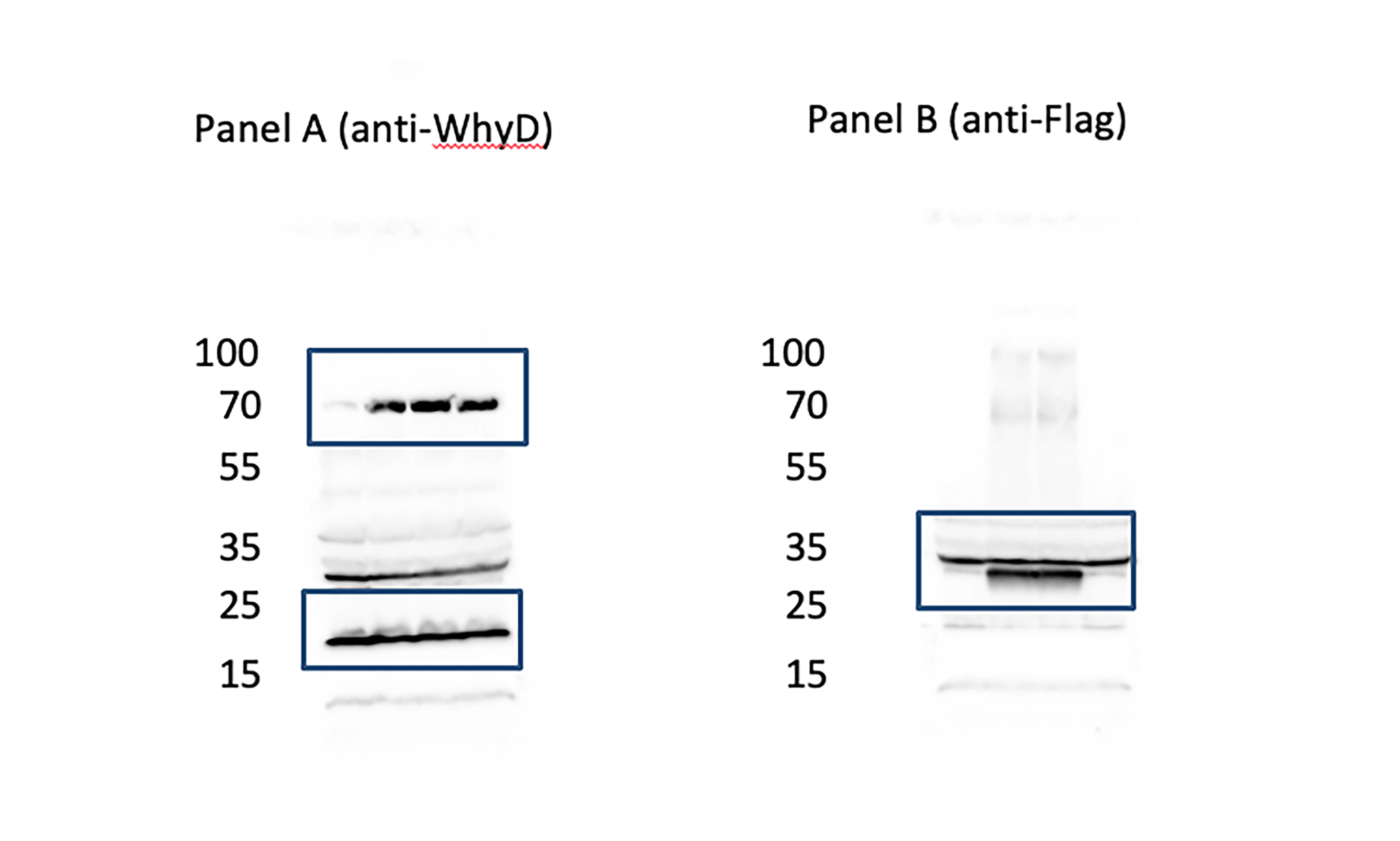

Supplement: Figure 2—figure supplement 1—source data 1. [file elife-76392-fig2-figsupp1-data1.zip › Figure 2 - figure supplement 1 - source data/Figure 2 - figure supplement 1- source data_abelled.tiff]

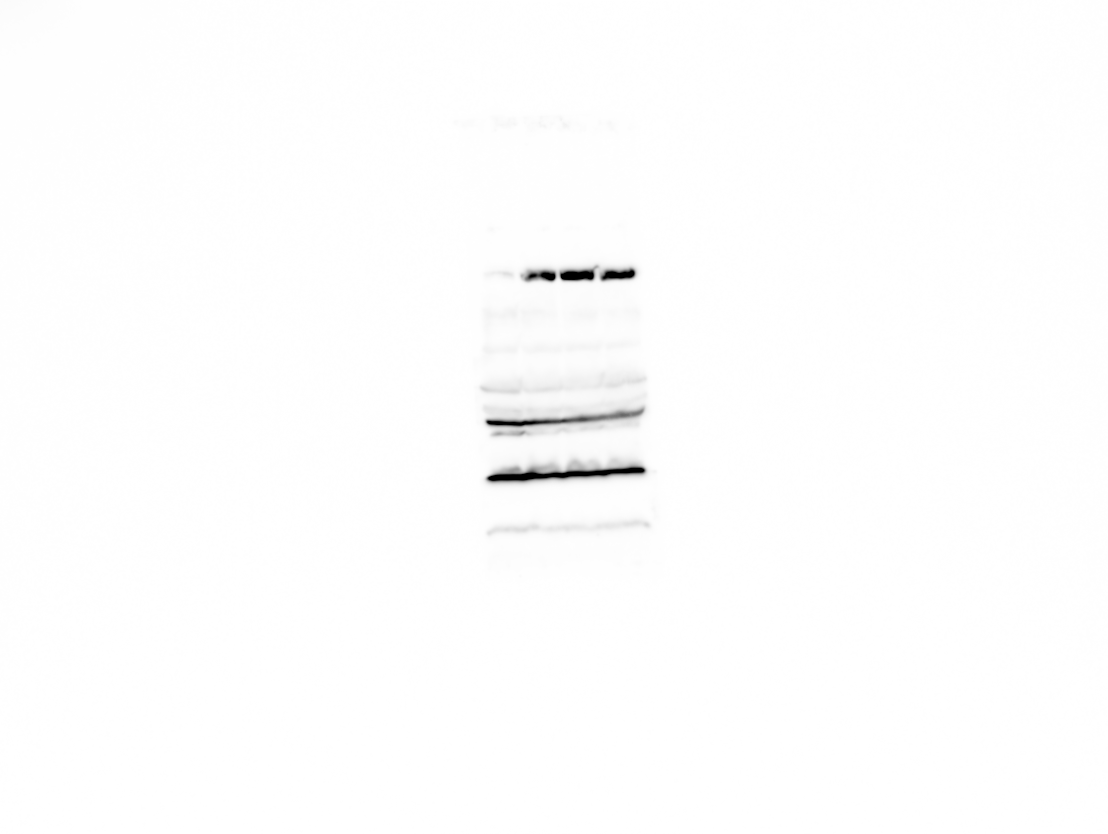

Supplement: Figure 2—figure supplement 1—source data 1. [file elife-76392-fig2-figsupp1-data1.zip › Figure 2 - figure supplement 1 - source data/Figure 2 - figure supplement 1 - source data_Panel A_anti-WhyD.tiff]

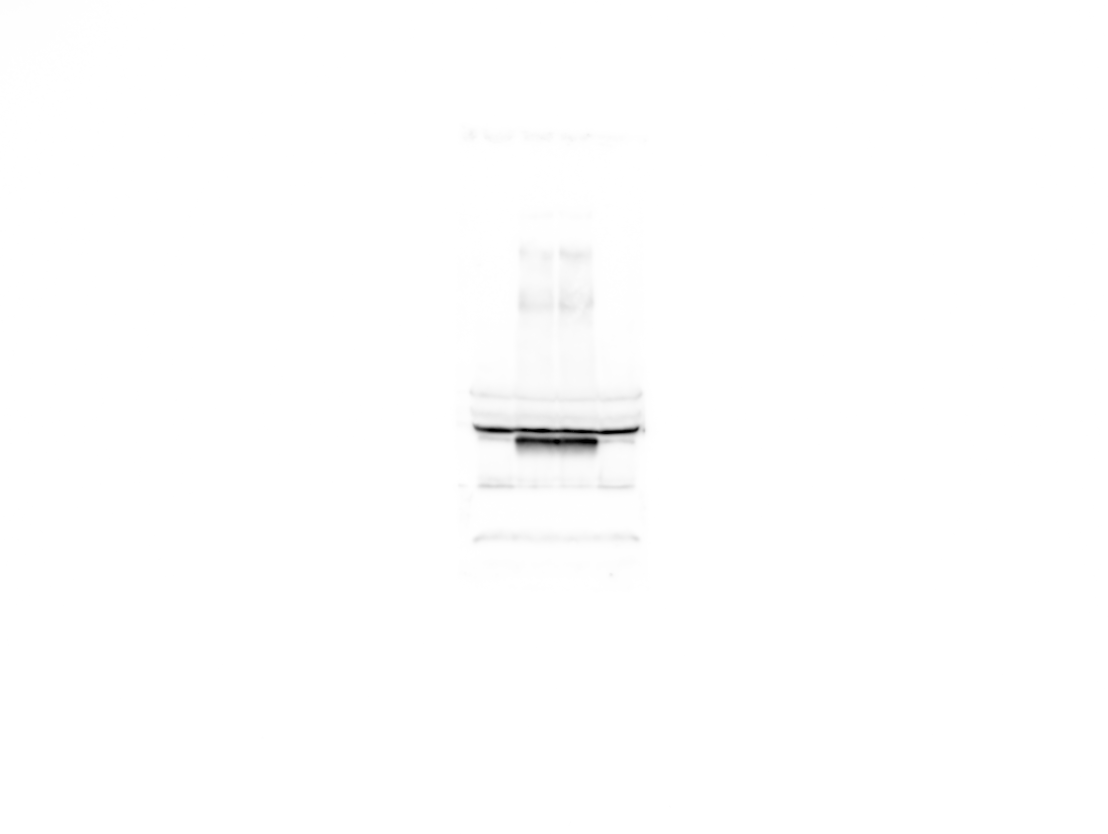

Supplement: Figure 2—figure supplement 1—source data 1. [file elife-76392-fig2-figsupp1-data1.zip › Figure 2 - figure supplement 1 - source data/Figure 2 - figure supplement 1 - source data_Panel B_anti-flag.tiff]

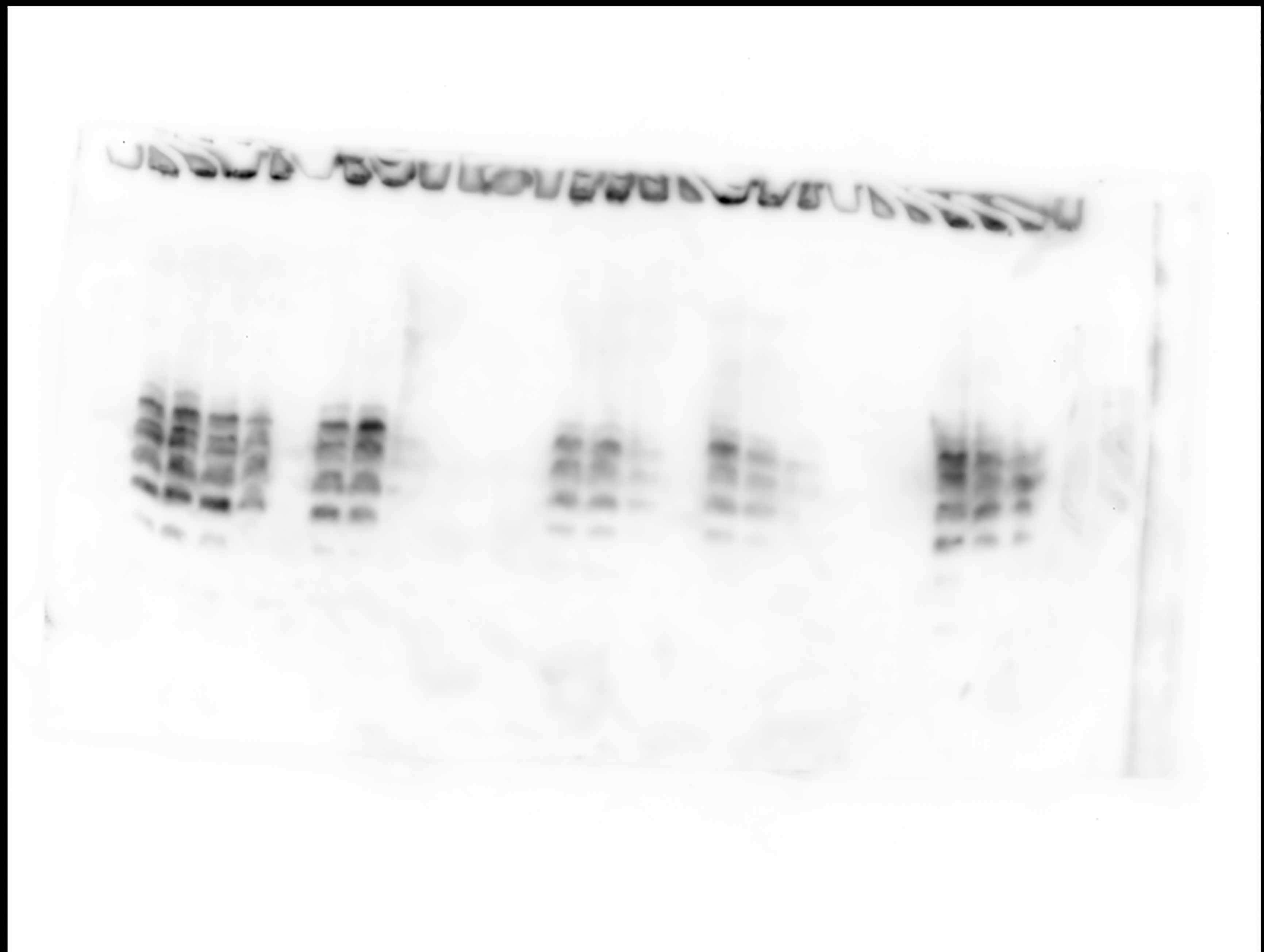

Supplement: Figure 2—figure supplement 2—source data 1. [file elife-76392-fig2-figsupp2-data1.zip › Figure 2 - figure supplement 2 - source data/ Figure 2 - figure supplement 2 - source data_LTAs.tiff]

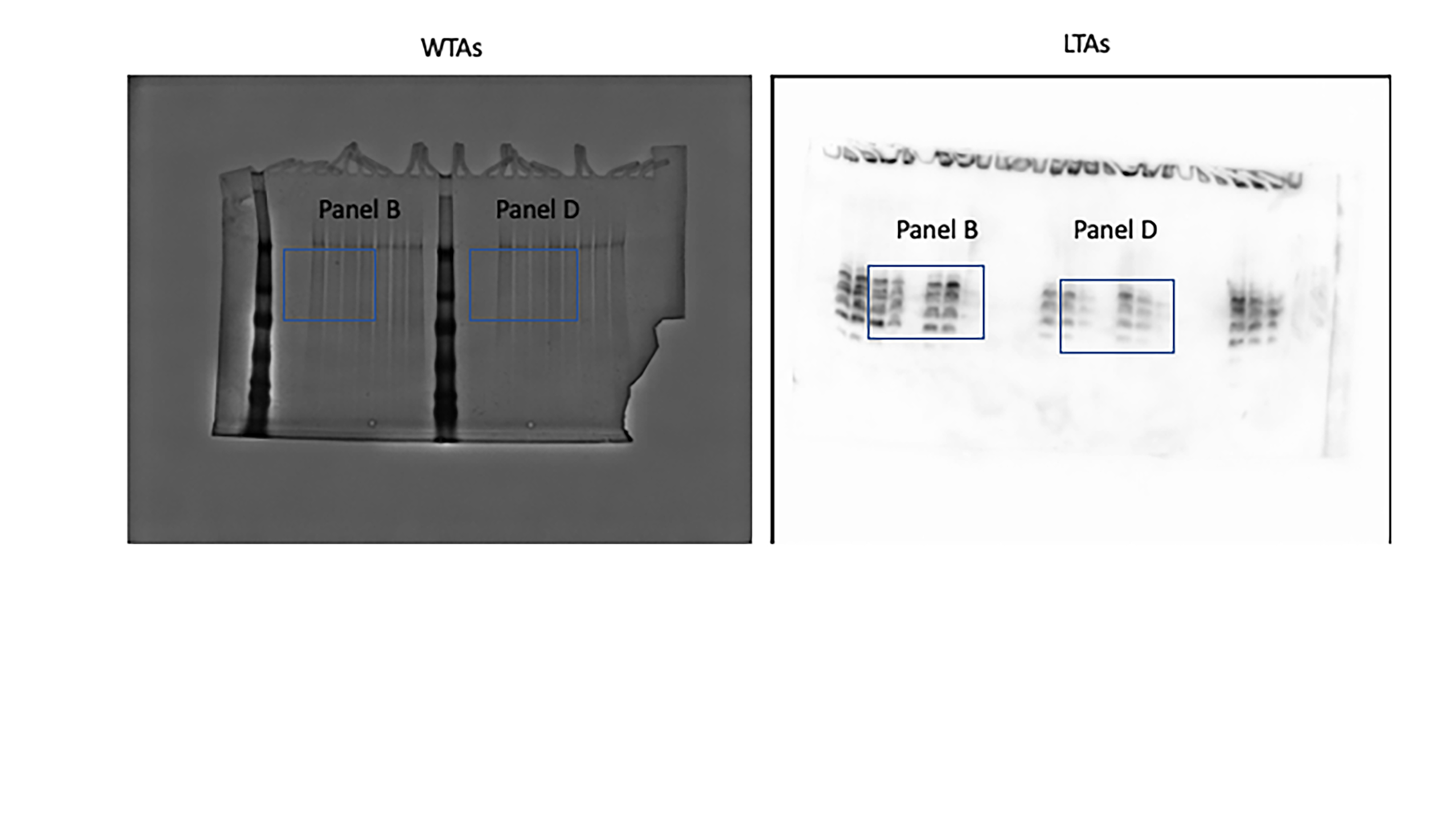

Supplement: Figure 2—figure supplement 2—source data 1. [file elife-76392-fig2-figsupp2-data1.zip › Figure 2 - figure supplement 2 - source data/Figure 2 - figure supplement 2 - source data_labeled.tiff]

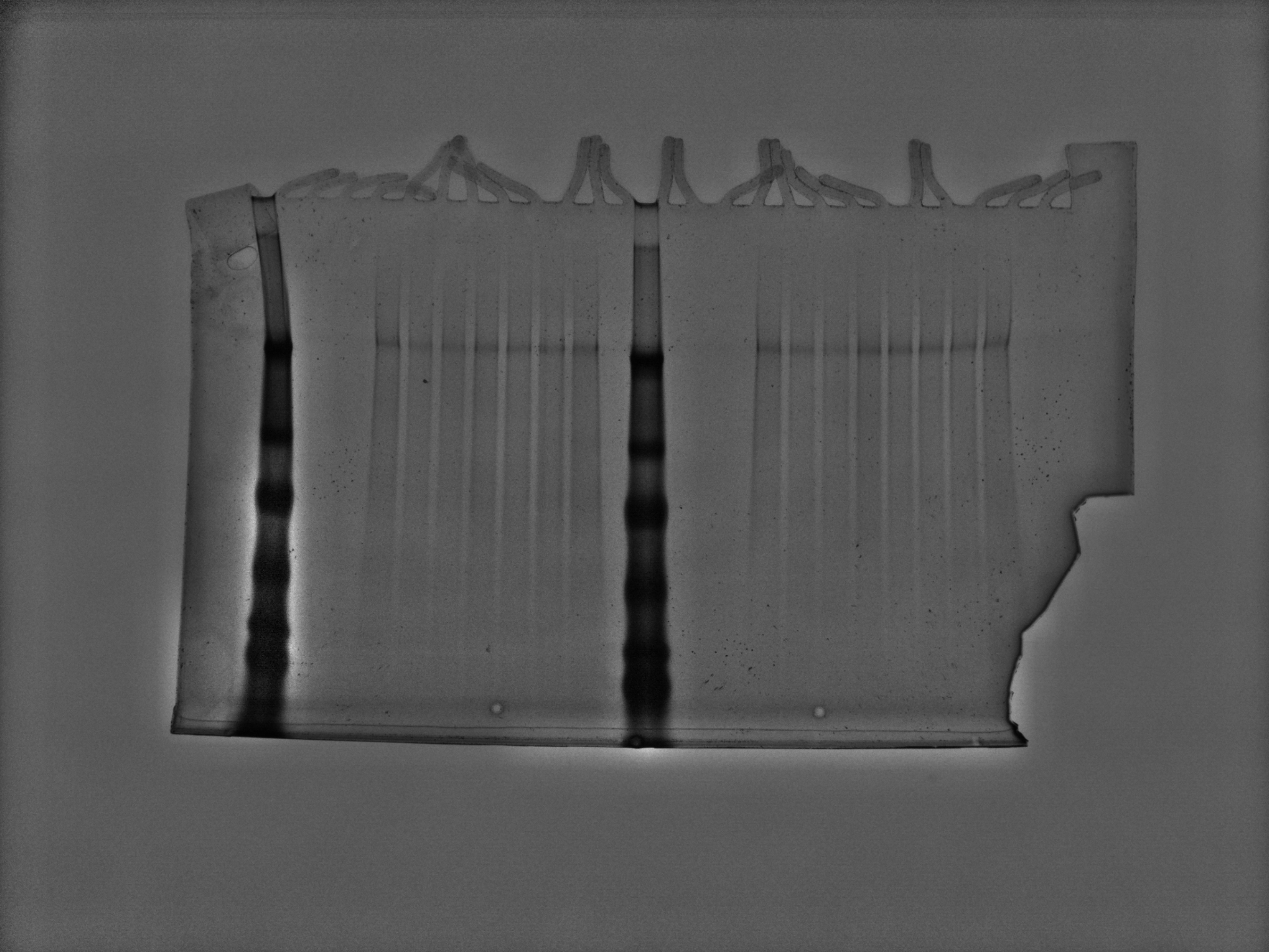

Supplement: Figure 2—figure supplement 2—source data 1. [file elife-76392-fig2-figsupp2-data1.zip › Figure 2 - figure supplement 2 - source data/Figure 2 - figure supplement 2 - source data_WTAs.tiff]

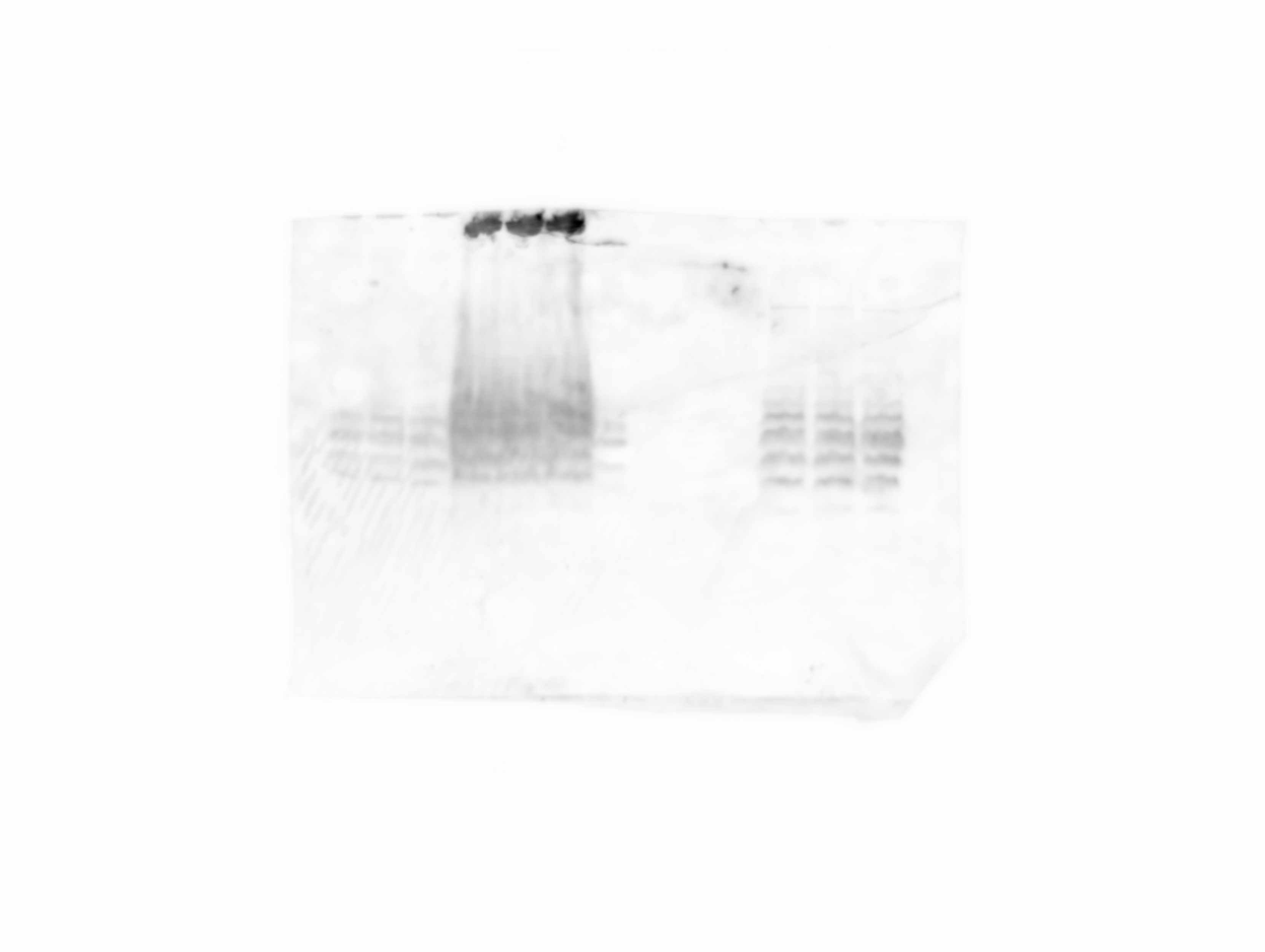

Supplement: Figure 3—source data 1. [file elife-76392-fig3-data1.zip › Figure 3 - source data/Figure 3 - source data_Panel 3C_LTAs.tiff]

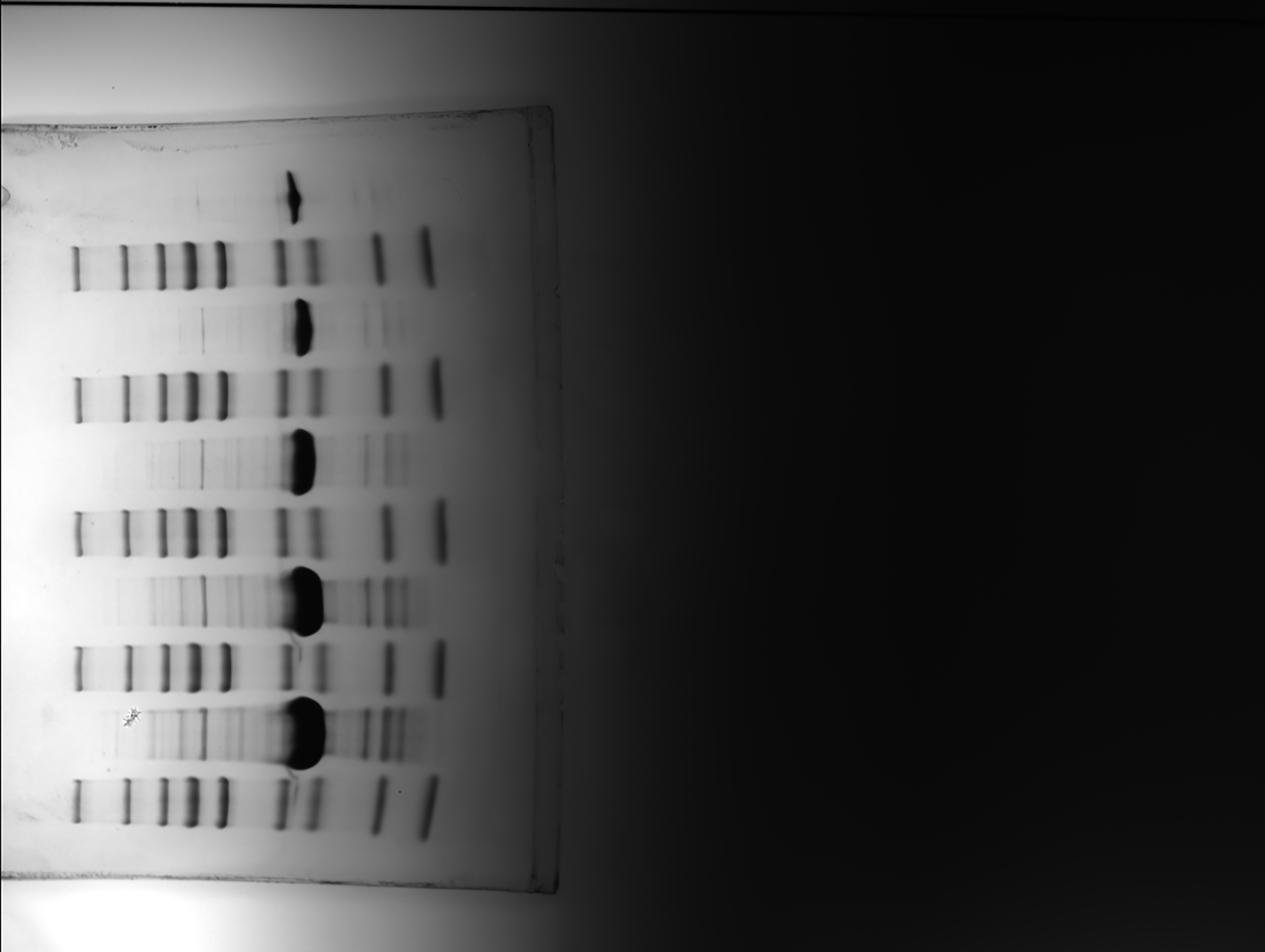

Supplement: Figure 3—source data 1. [file elife-76392-fig3-data1.zip › Figure 3 - source data/Figure 3 - source data_Panel 3B_WhyDCT.tiff]

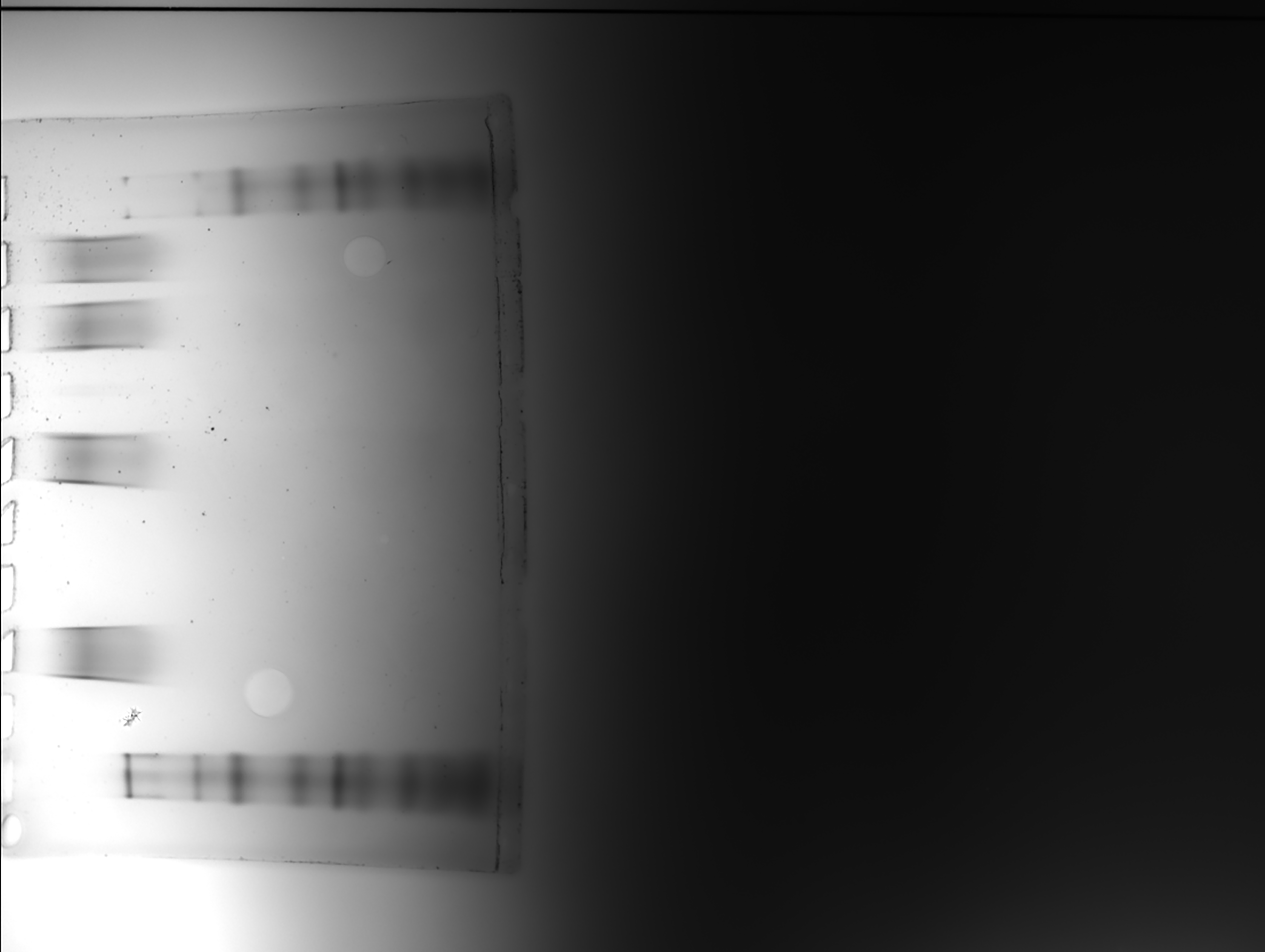

Supplement: Figure 3—source data 1. [file elife-76392-fig3-data1.zip › Figure 3 - source data/Figure 3 - source data_Panel 3B_WTA.tiff]

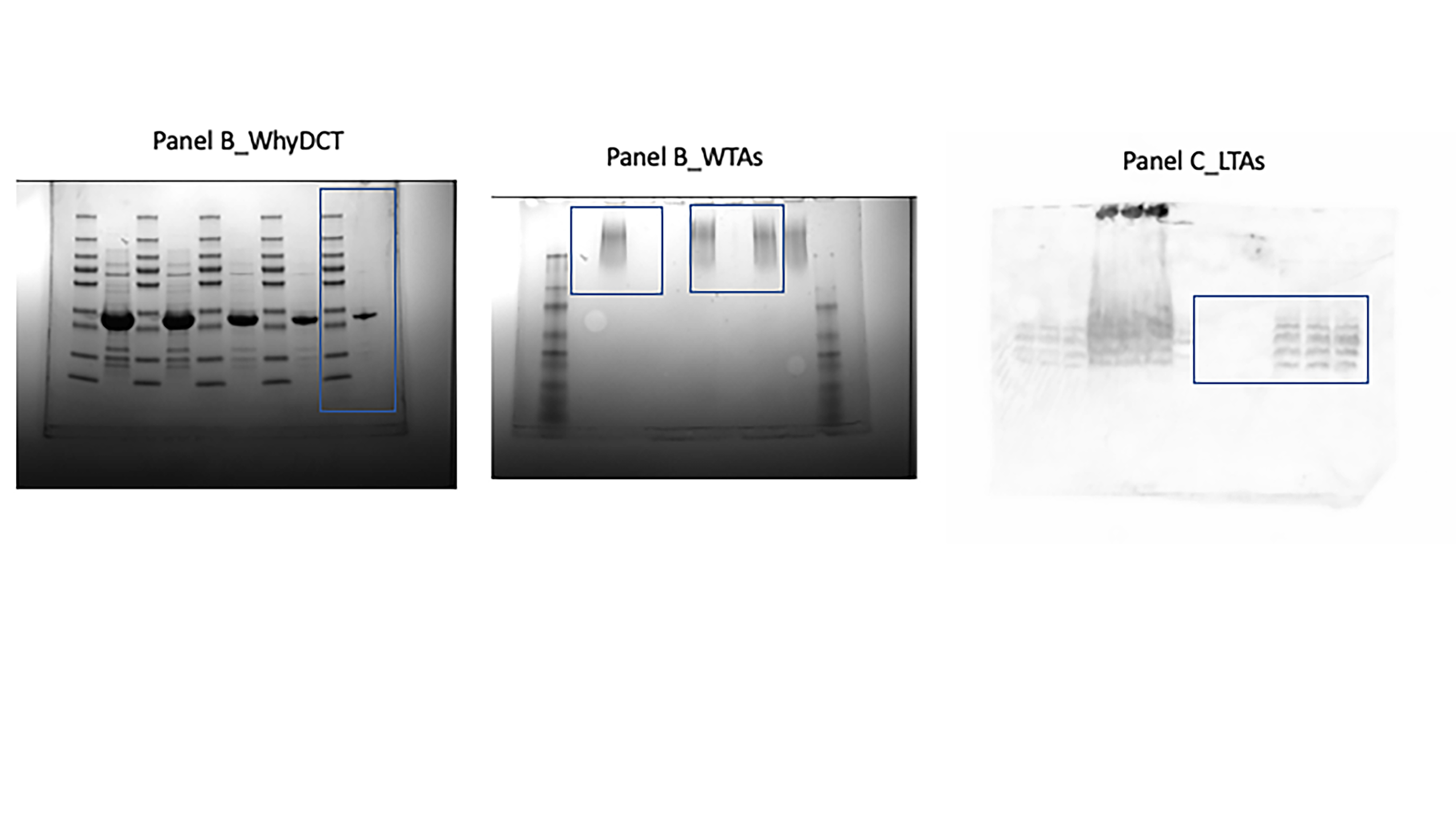

Supplement: Figure 3—source data 1. [file elife-76392-fig3-data1.zip › Figure 3 - source data/Figure 3 - source data_labeled.tiff]

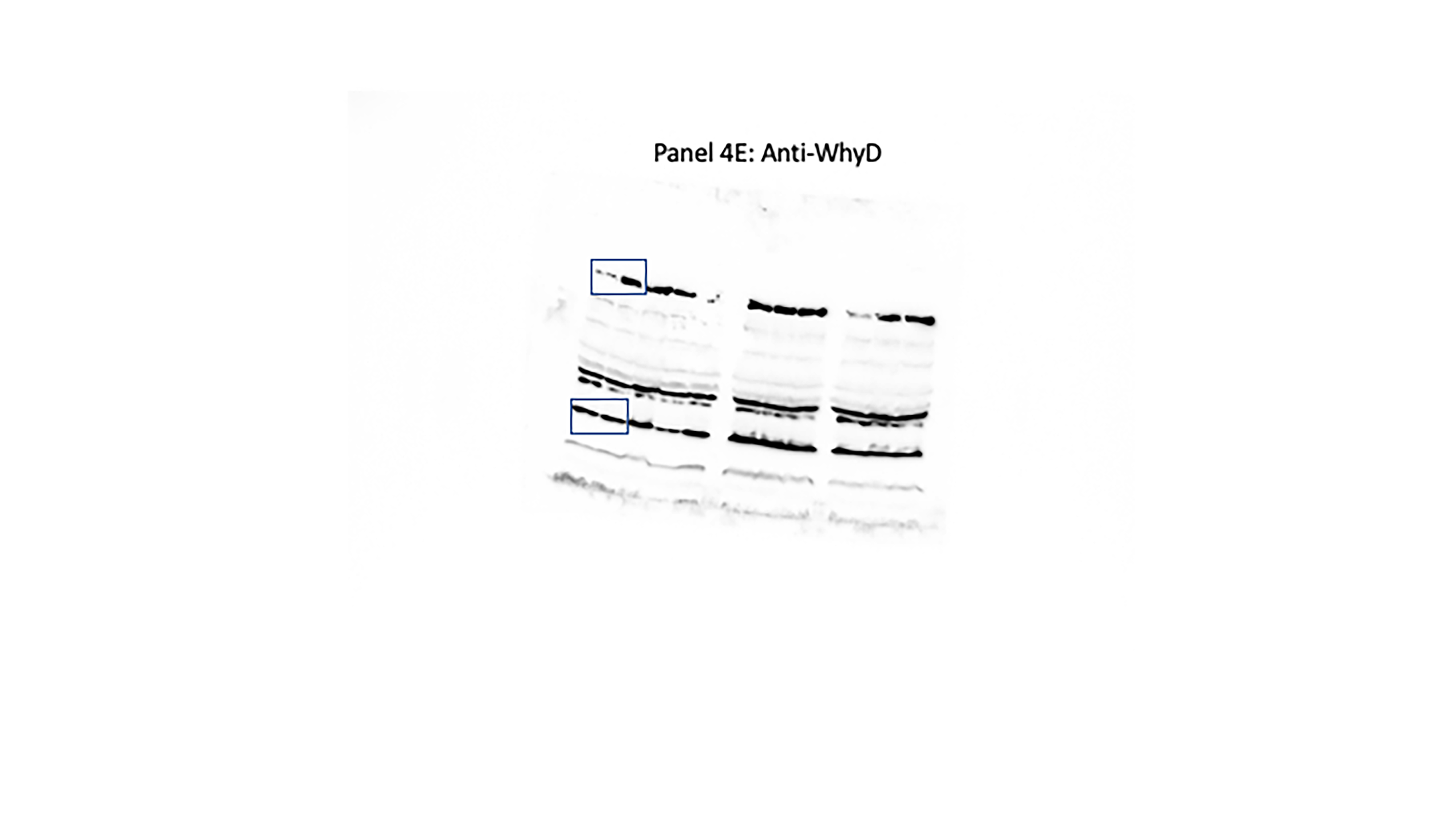

Supplement: Figure 4—source data 3. [file elife-76392-fig4-data3.zip › Figure 4E - source data/Figure 4E - source data_WhyD_labeled.tiff]

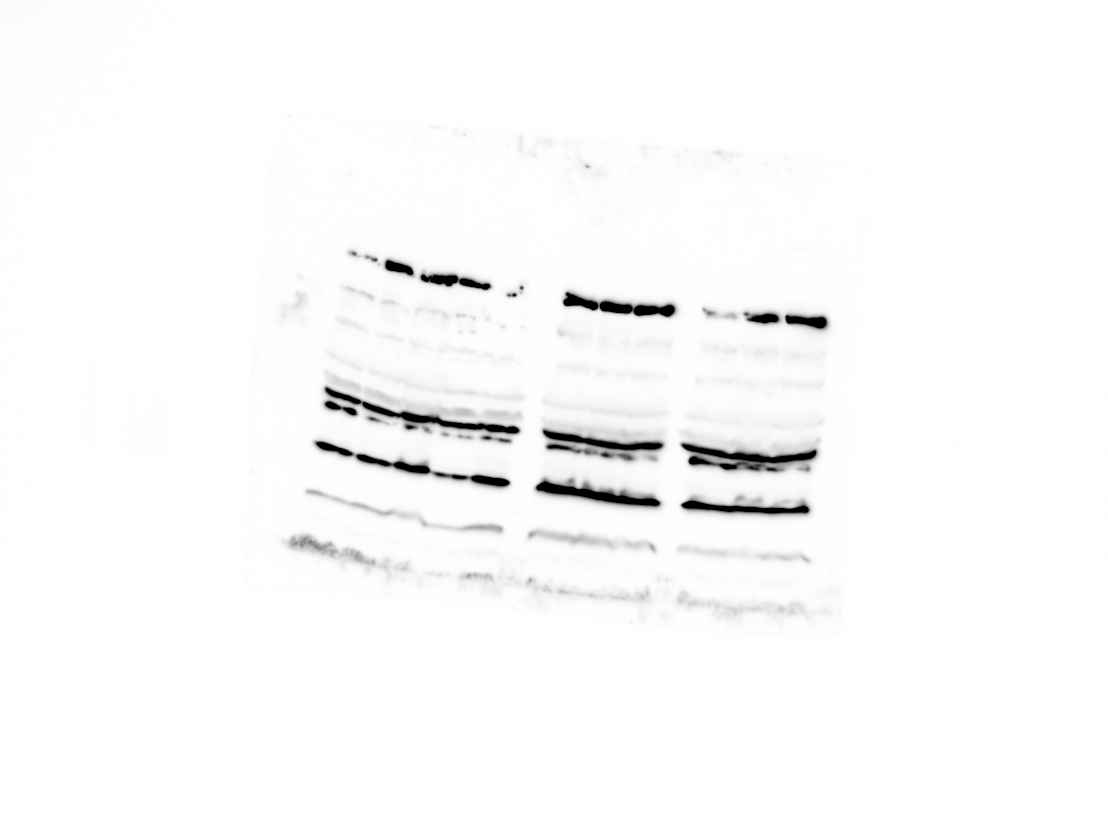

Supplement: Figure 4—source data 3. [file elife-76392-fig4-data3.zip › Figure 4E - source data/Figure 4E - source data_WhyD.tif]

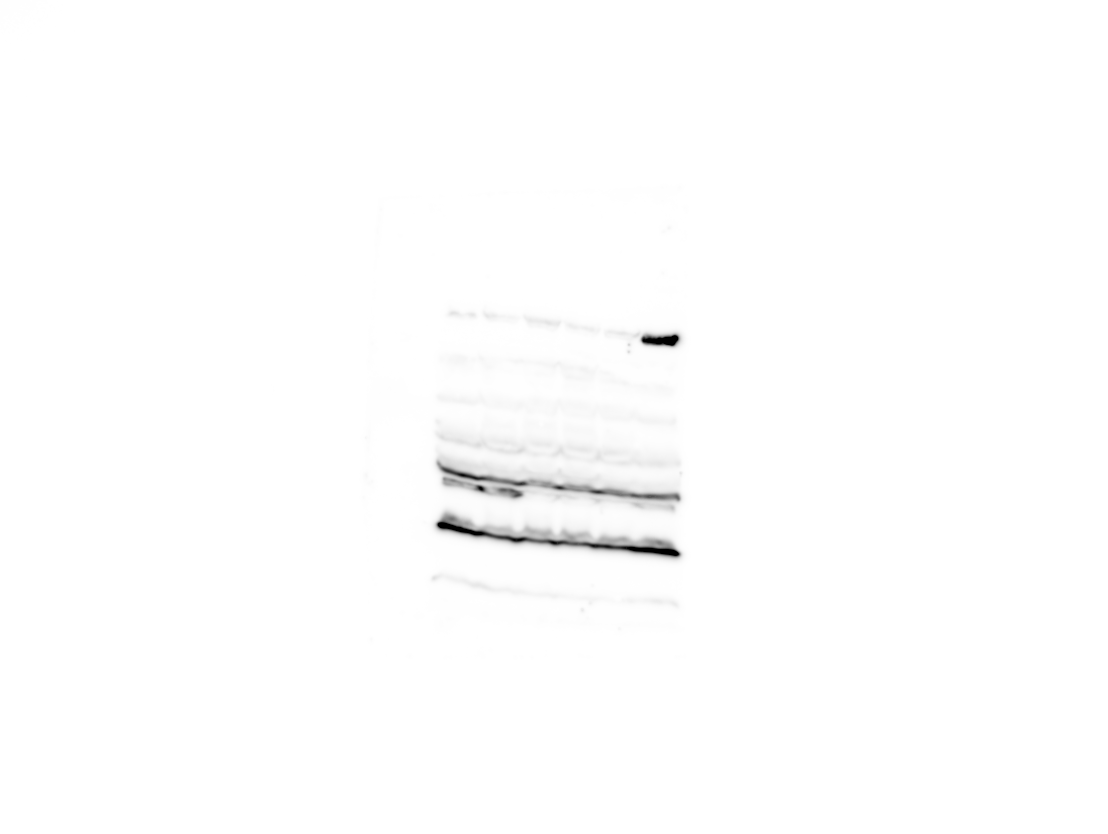

Supplement: Figure 4—figure supplement 3—source data 1. [file elife-76392-fig4-figsupp3-data1.zip › Figure 4 figure supplement 3 - source data/Figure 4 - figure supplement 3-source data_whyD.tif]

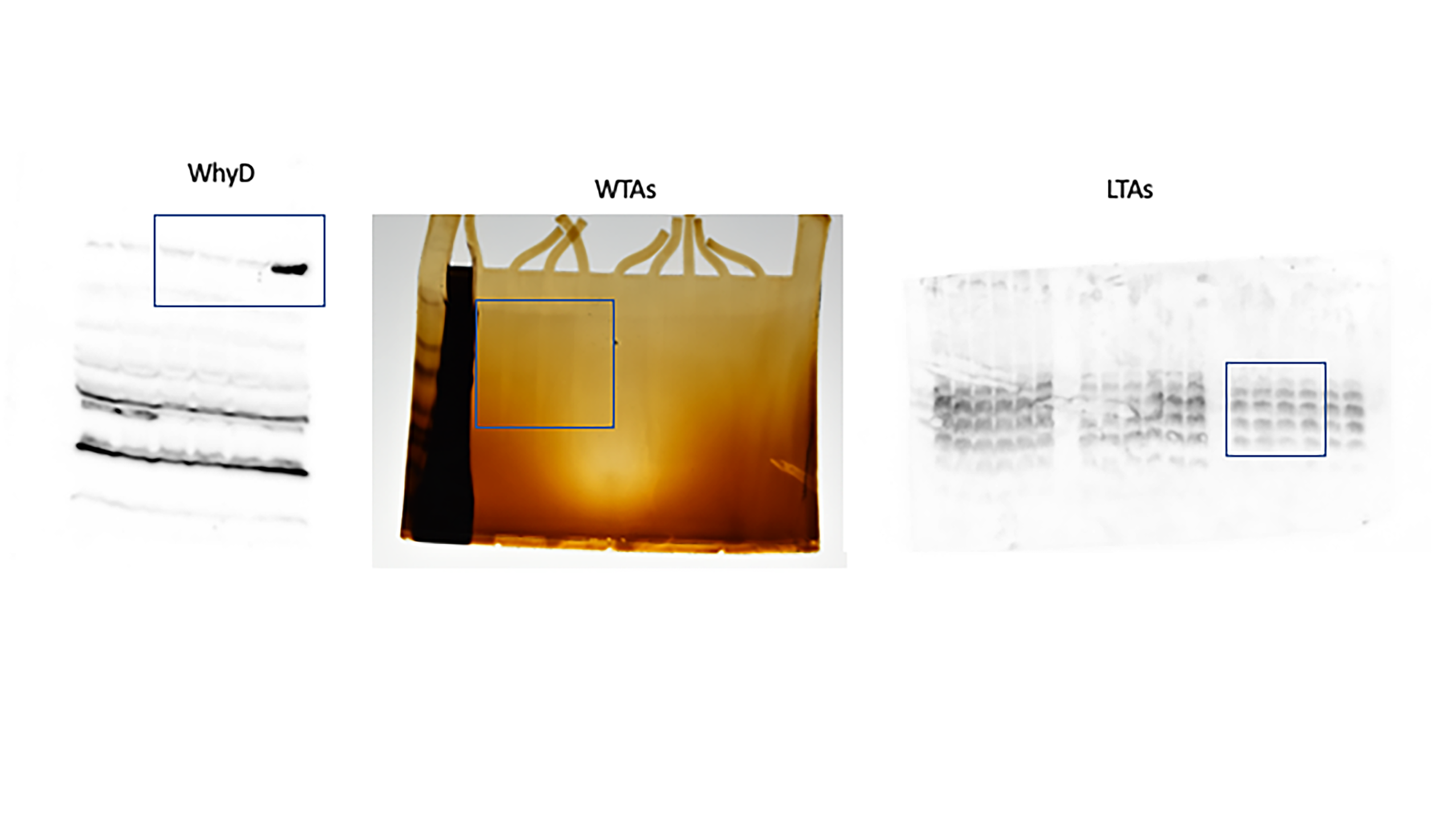

Supplement: Figure 4—figure supplement 3—source data 1. [file elife-76392-fig4-figsupp3-data1.zip › Figure 4 figure supplement 3 - source data/Figure 4 - figure supplement 3-source data.tiff]

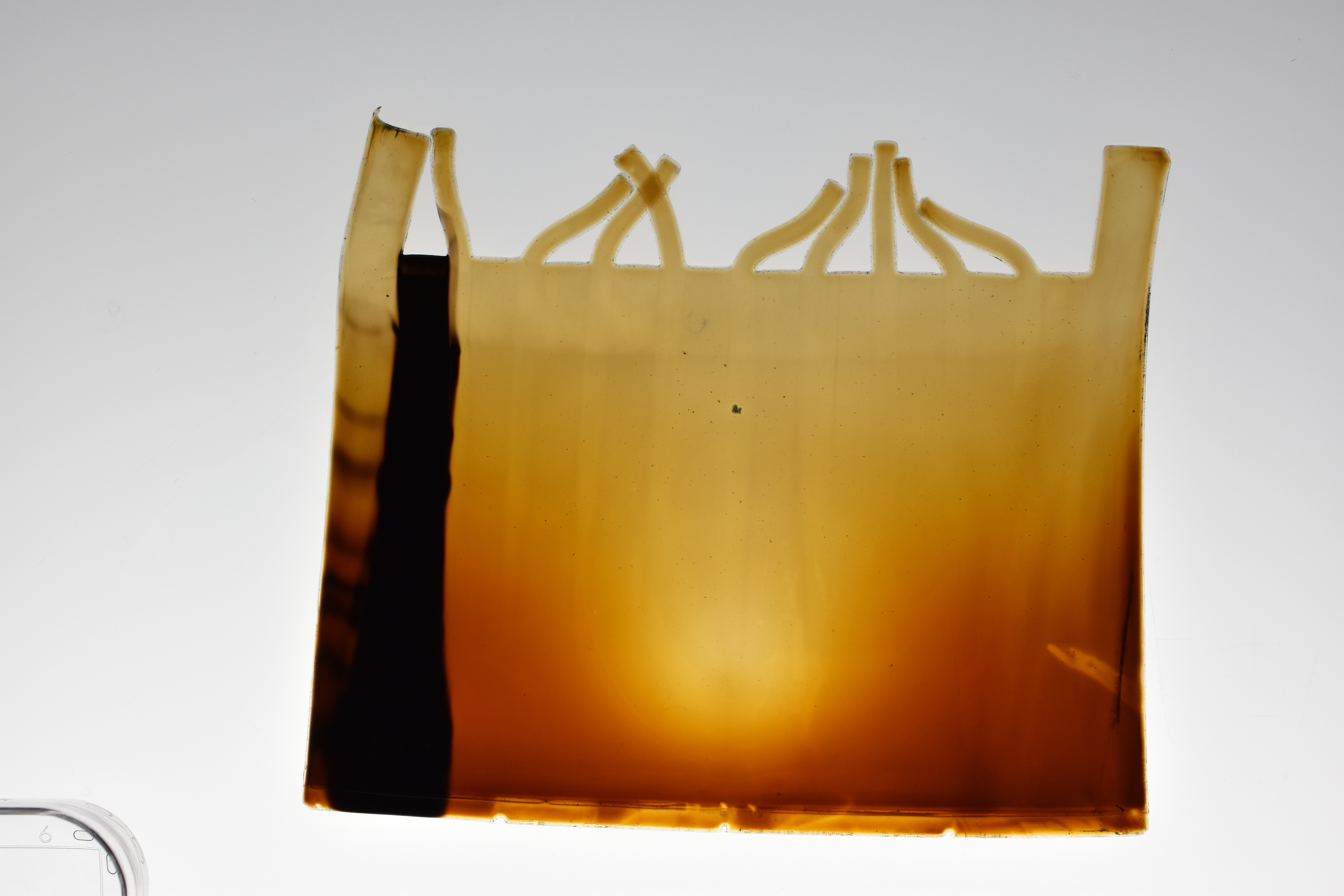

Supplement: Figure 4—figure supplement 3—source data 1. [file elife-76392-fig4-figsupp3-data1.zip › Figure 4 figure supplement 3 - source data/Figure 4 - figure supplement 3-source data_WTA.tiff]

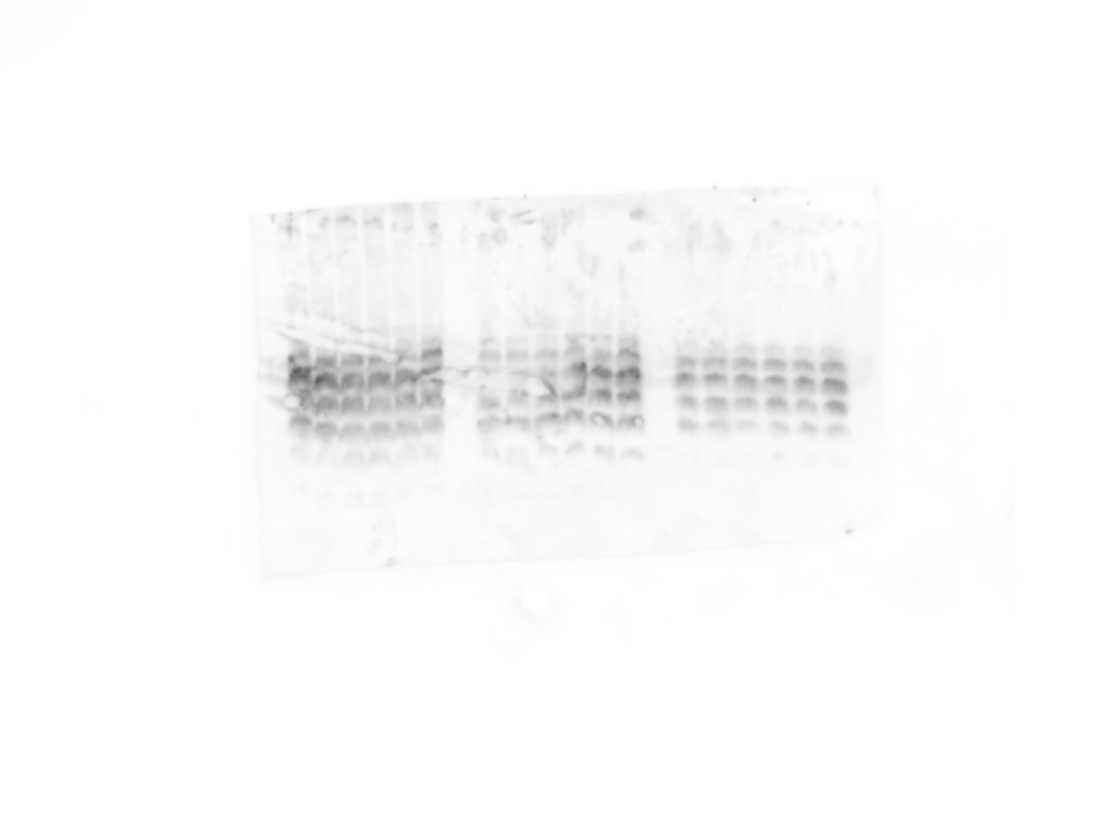

Supplement: Figure 4—figure supplement 3—source data 1. [file elife-76392-fig4-figsupp3-data1.zip › Figure 4 figure supplement 3 - source data/Figure 4 - figure supplement 3-source data_LTA.tif]

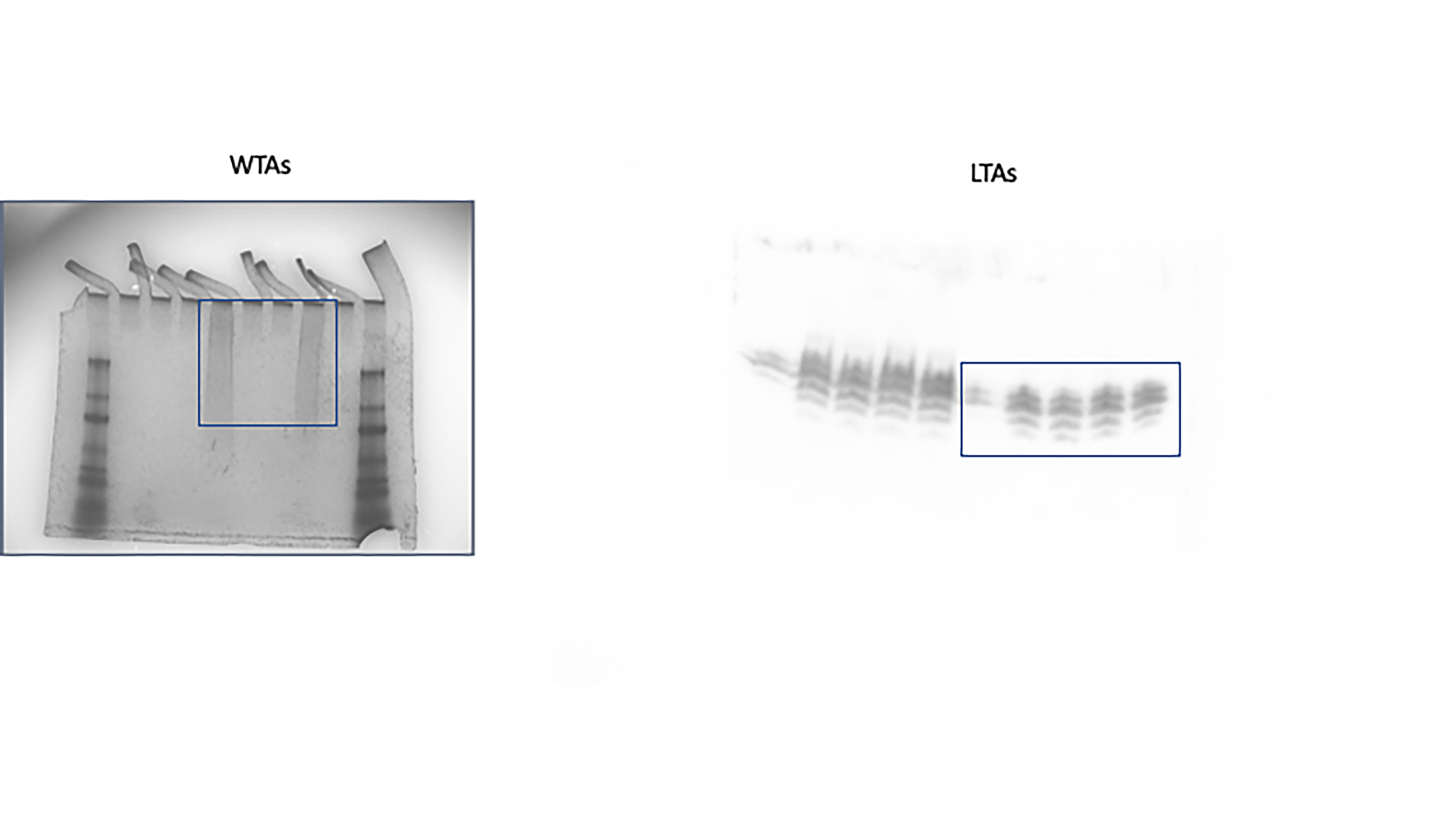

Supplement: Figure 5—source data 1. [file elife-76392-fig5-data1.zip › Figure 5 - source data/Figure 5C - source data/Figure 5C - source data_labeled.tiff]

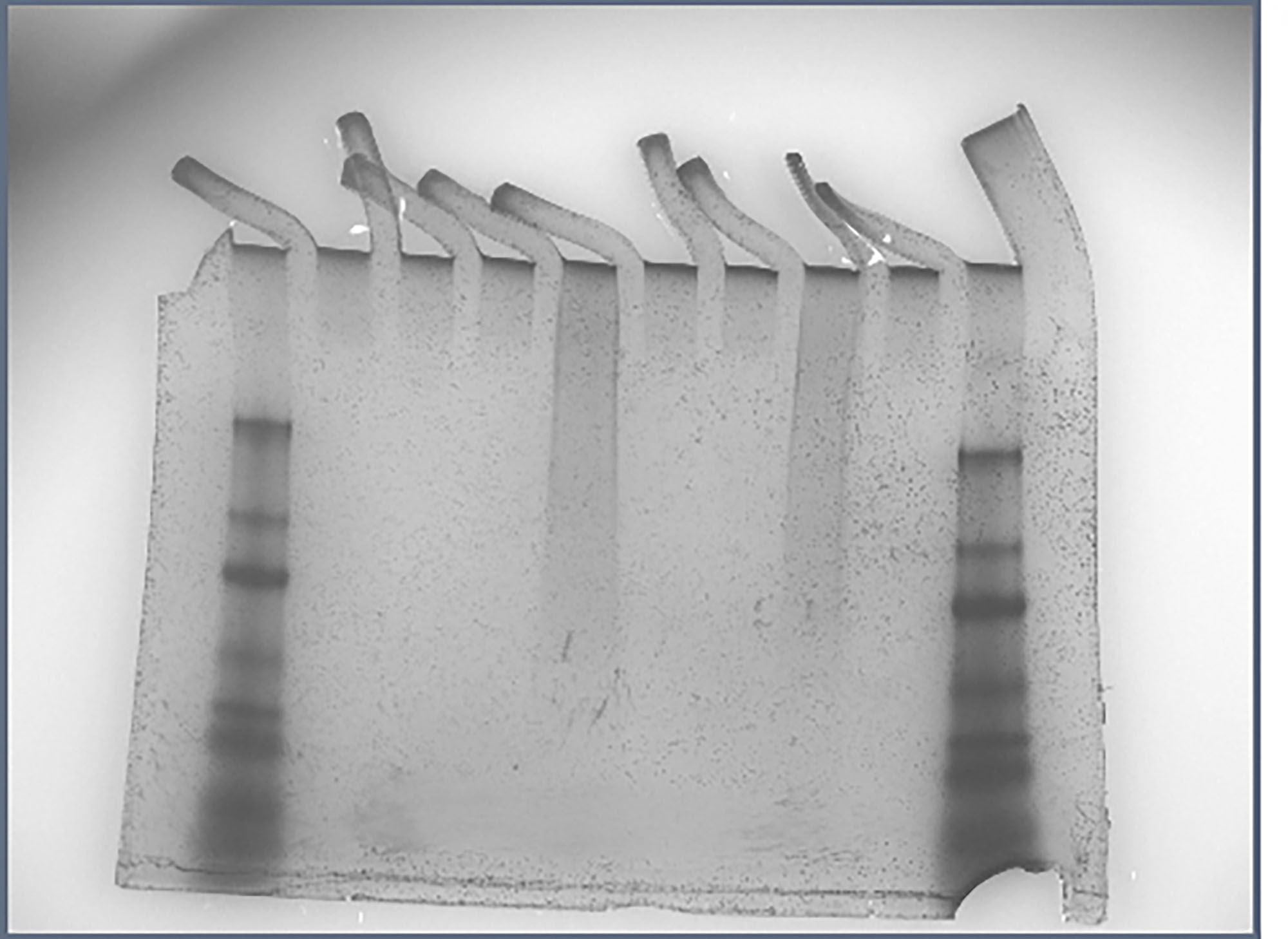

Supplement: Figure 5—source data 1. [file elife-76392-fig5-data1.zip › Figure 5 - source data/Figure 5C - source data/Figure 5C - source data_WTA.tiff]

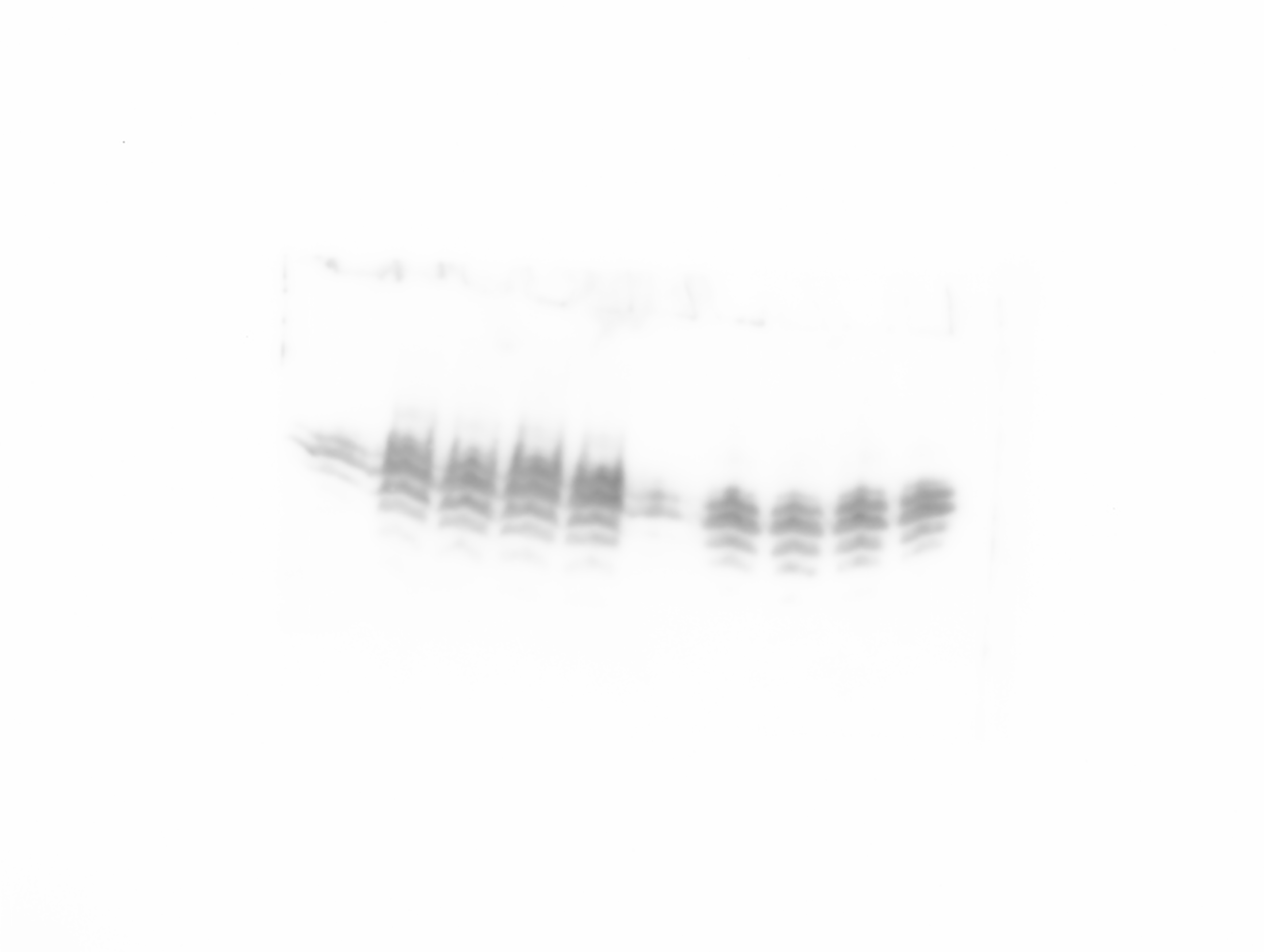

Supplement: Figure 5—source data 1. [file elife-76392-fig5-data1.zip › Figure 5 - source data/Figure 5C - source data/Figure 5C - source data_LTA.tiff]

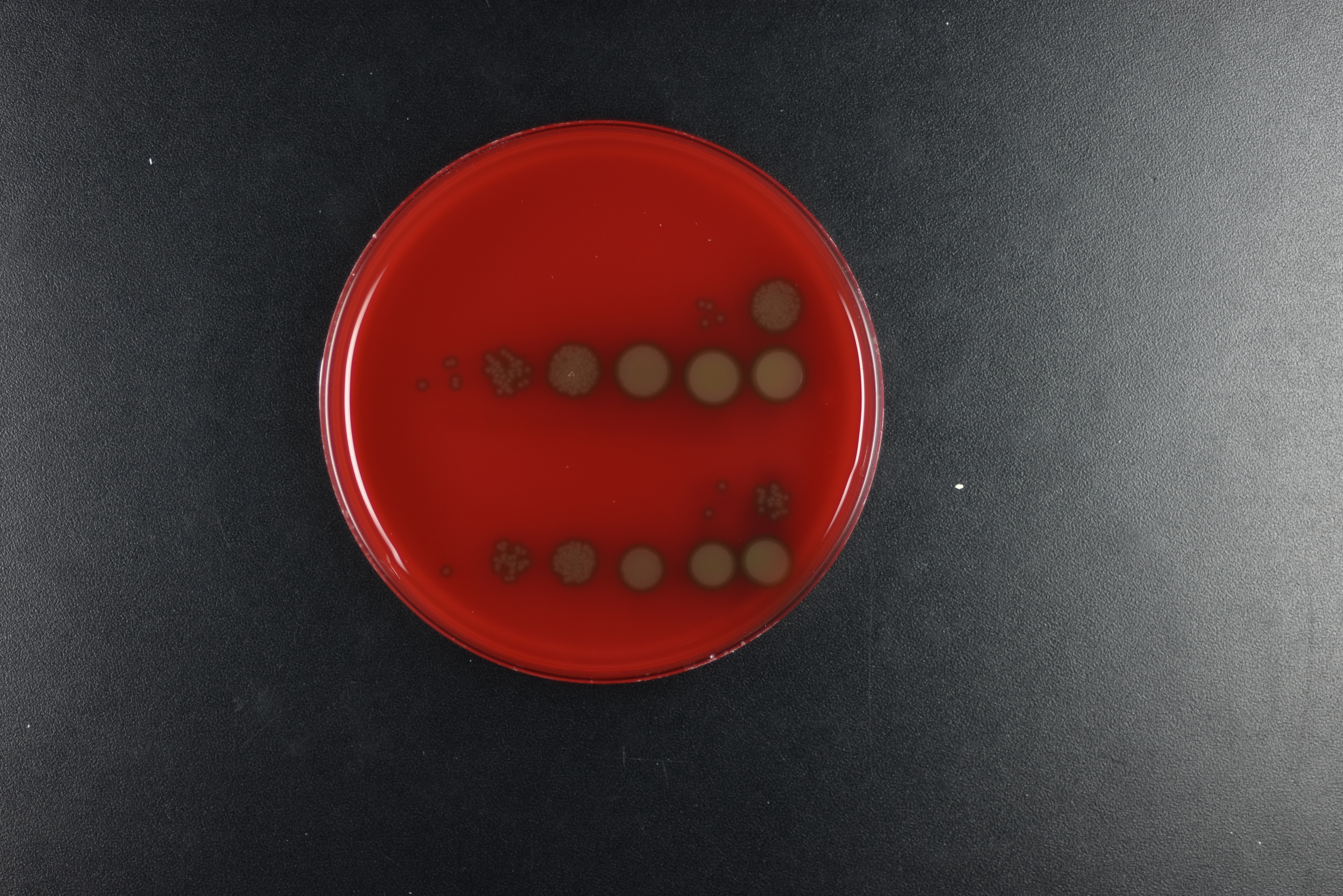

Supplement: Figure 6—figure supplement 1—source data 1. [file elife-76392-fig6-figsupp1-data1.zip › Figure 6 - figure supplement 1A- source data/Figure 6 - figure supplement 1A - source data_-Zn.tiff]

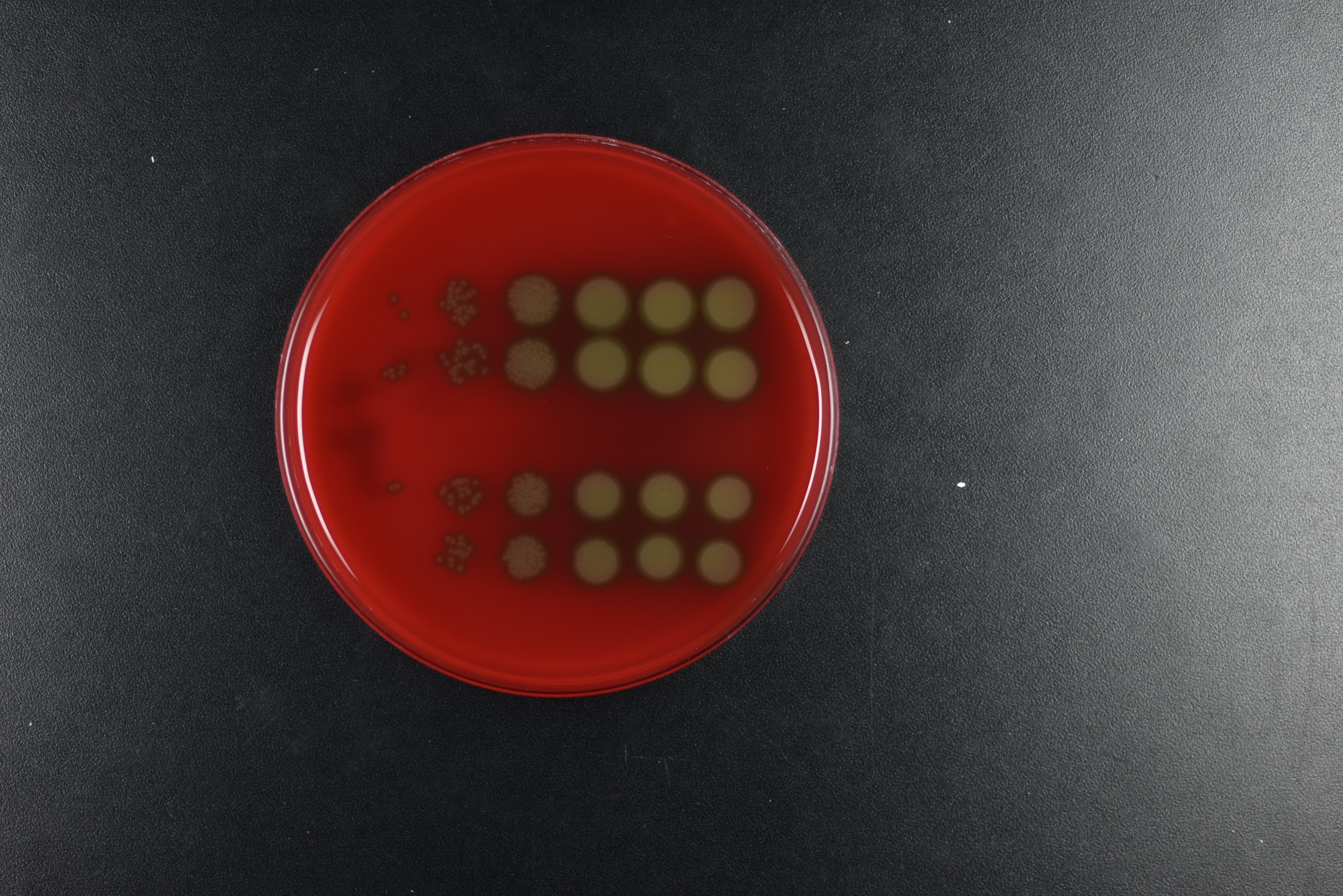

Supplement: Figure 6—figure supplement 1—source data 1. [file elife-76392-fig6-figsupp1-data1.zip › Figure 6 - figure supplement 1A- source data/Figure 6 - figure supplement 1A - source data_+Zn.tiff]

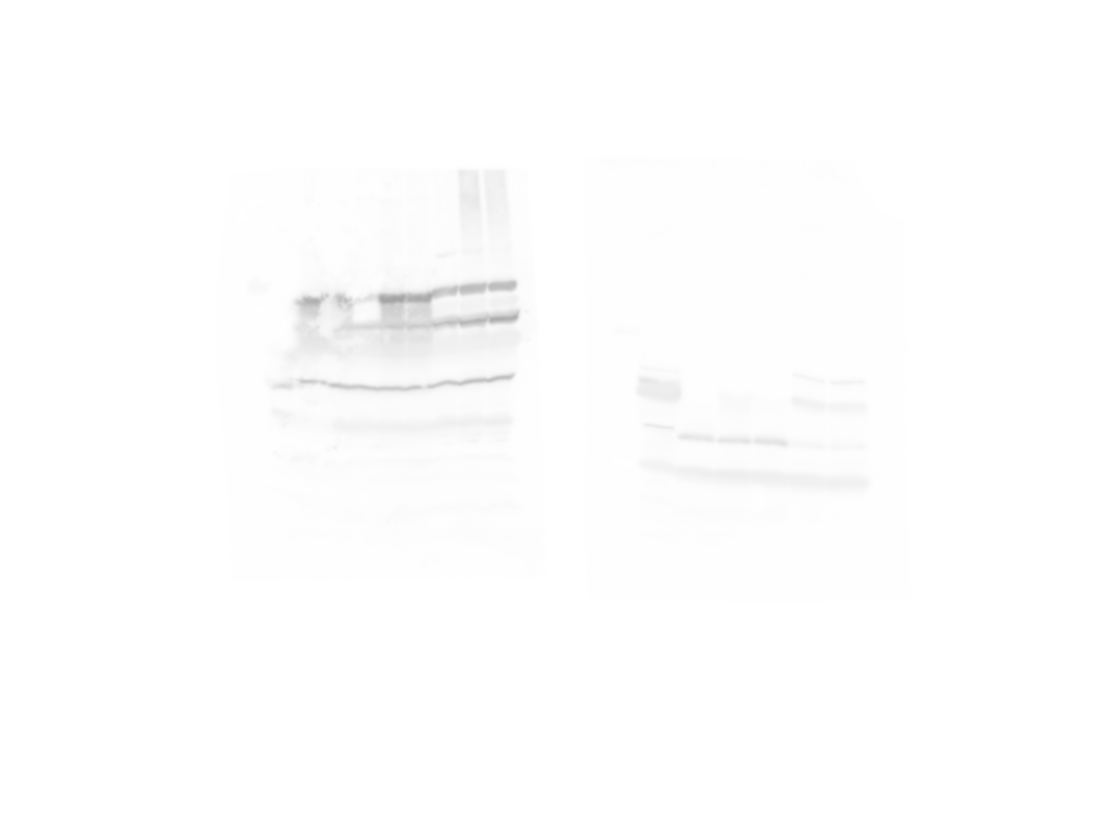

Supplement: Figure 6—figure supplement 1—source data 2. [file elife-76392-fig6-figsupp1-data2.zip › Figure 6 - figure supplement 1B - source data/Figure 6 - figure supplement 1B - source data_lWhyD.tiff]

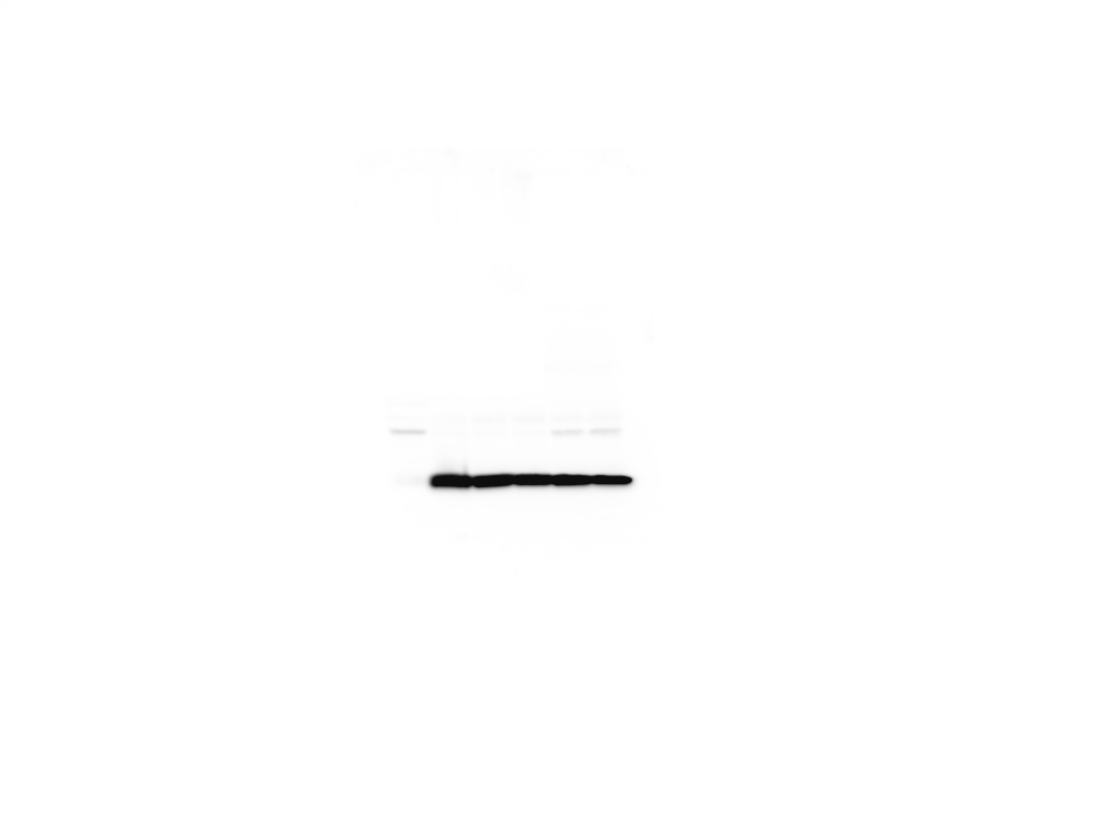

Supplement: Figure 6—figure supplement 1—source data 2. [file elife-76392-fig6-figsupp1-data2.zip › Figure 6 - figure supplement 1B - source data/Figure 6 - figure supplement 1B - source data_GFP.tiff]

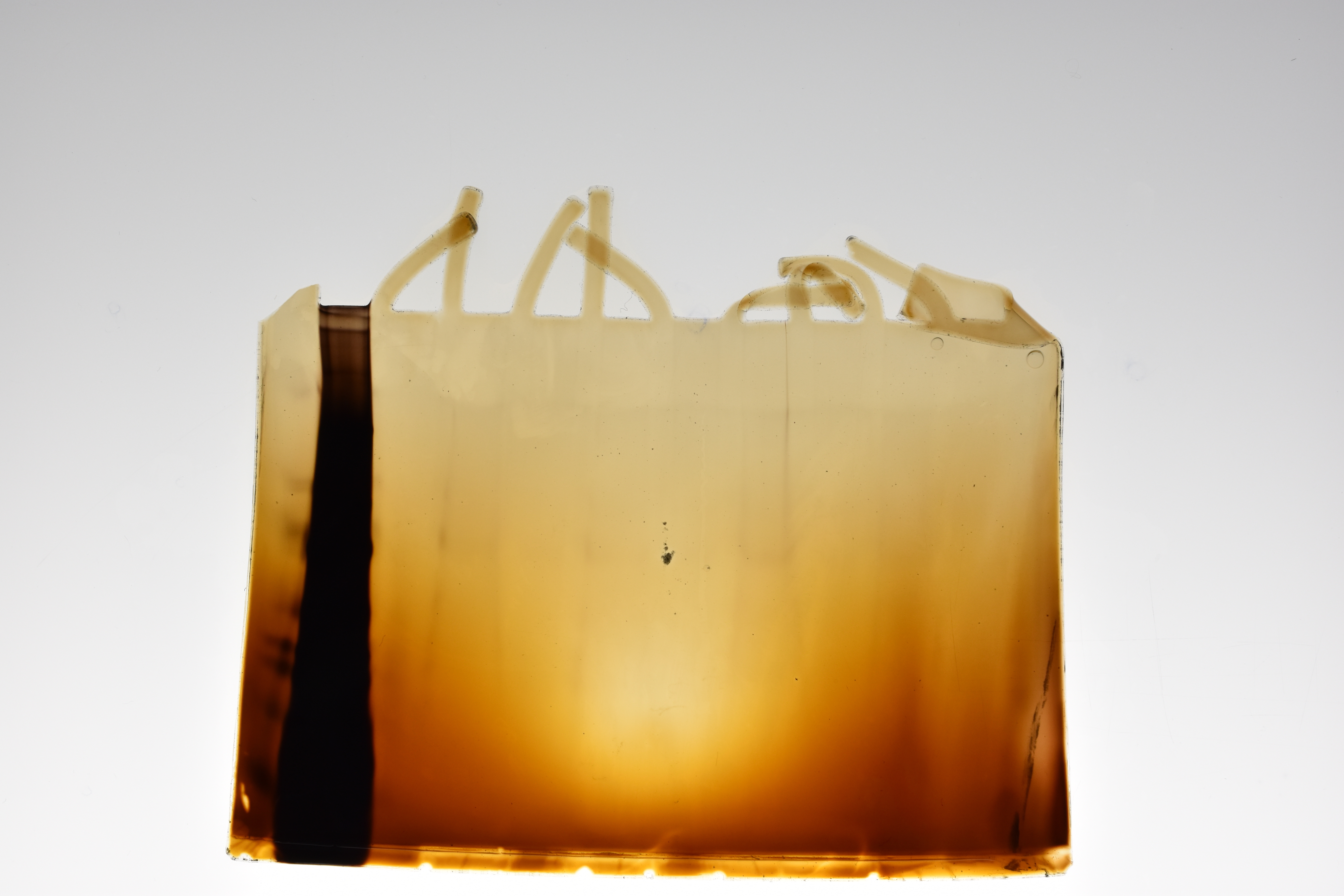

Supplement: Figure 7—figure supplement 3—source data 1. [file elife-76392-fig7-figsupp3-data1.zip › Figure 7 - figure supplement 3 - source data/Figure 7 - figure supplement 3 - source data_WTA.tiff]

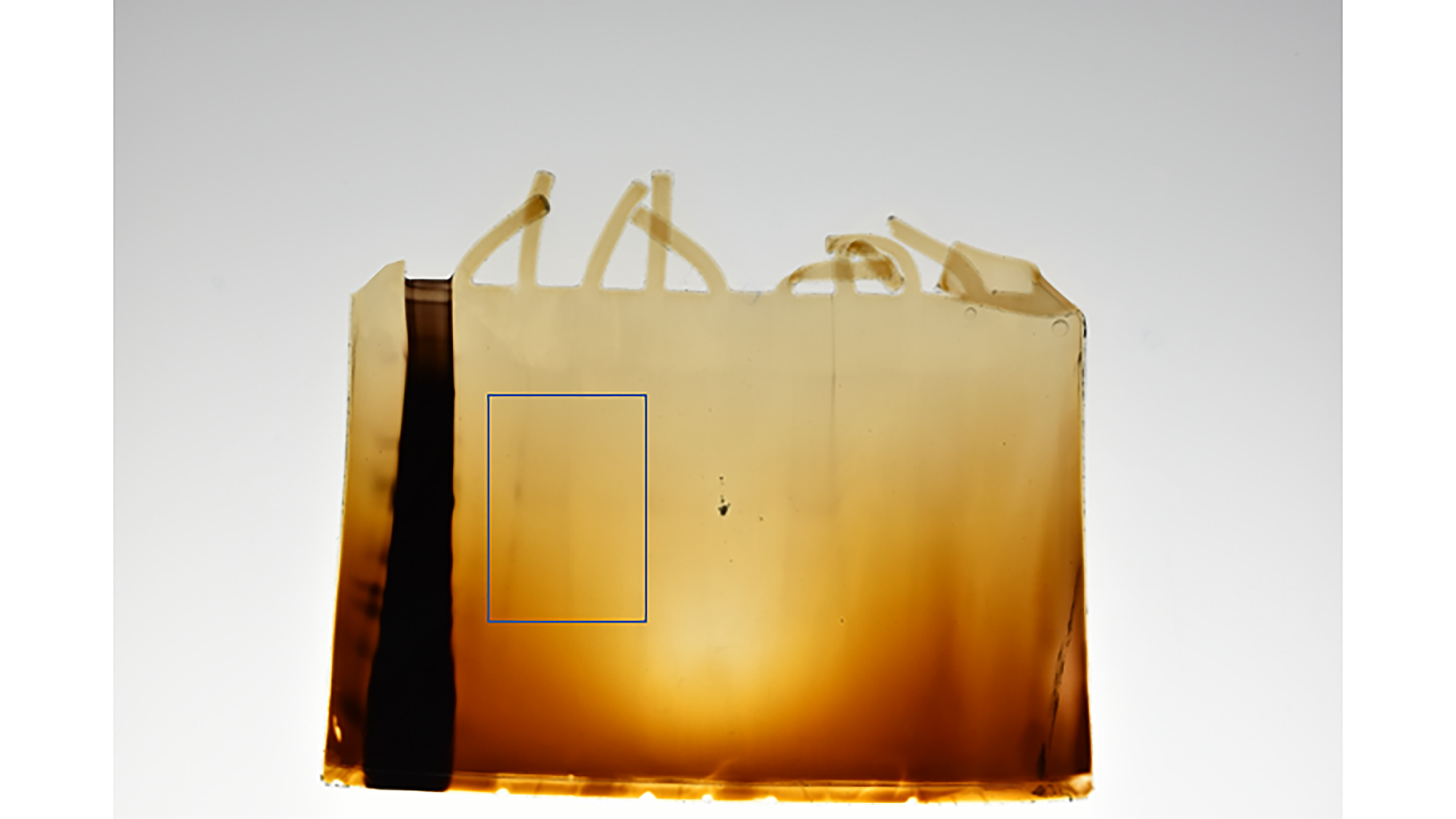

Supplement: Figure 7—figure supplement 3—source data 1. [file elife-76392-fig7-figsupp3-data1.zip › Figure 7 - figure supplement 3 - source data/Figure 7 - figure supplement 3 - source data_WTA_labeled.tiff]

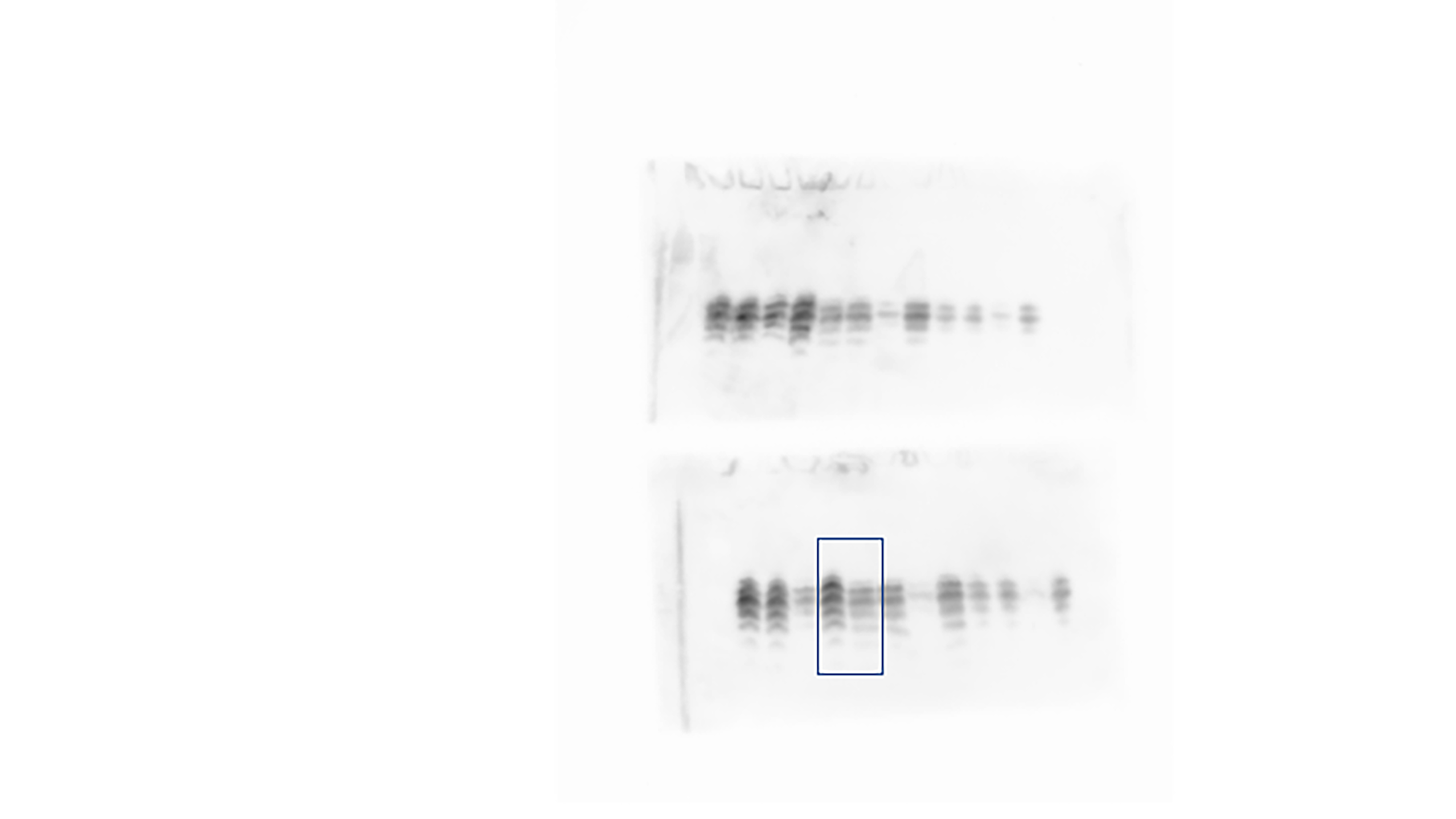

Supplement: Figure 8—figure supplement 1—source data 1. [file elife-76392-fig8-figsupp1-data1.zip › Figure 8 - figure suuplement 1 - source data/Figure 8 figure supplement 1 - source data_WTA_choline blot_labeled.tiff]

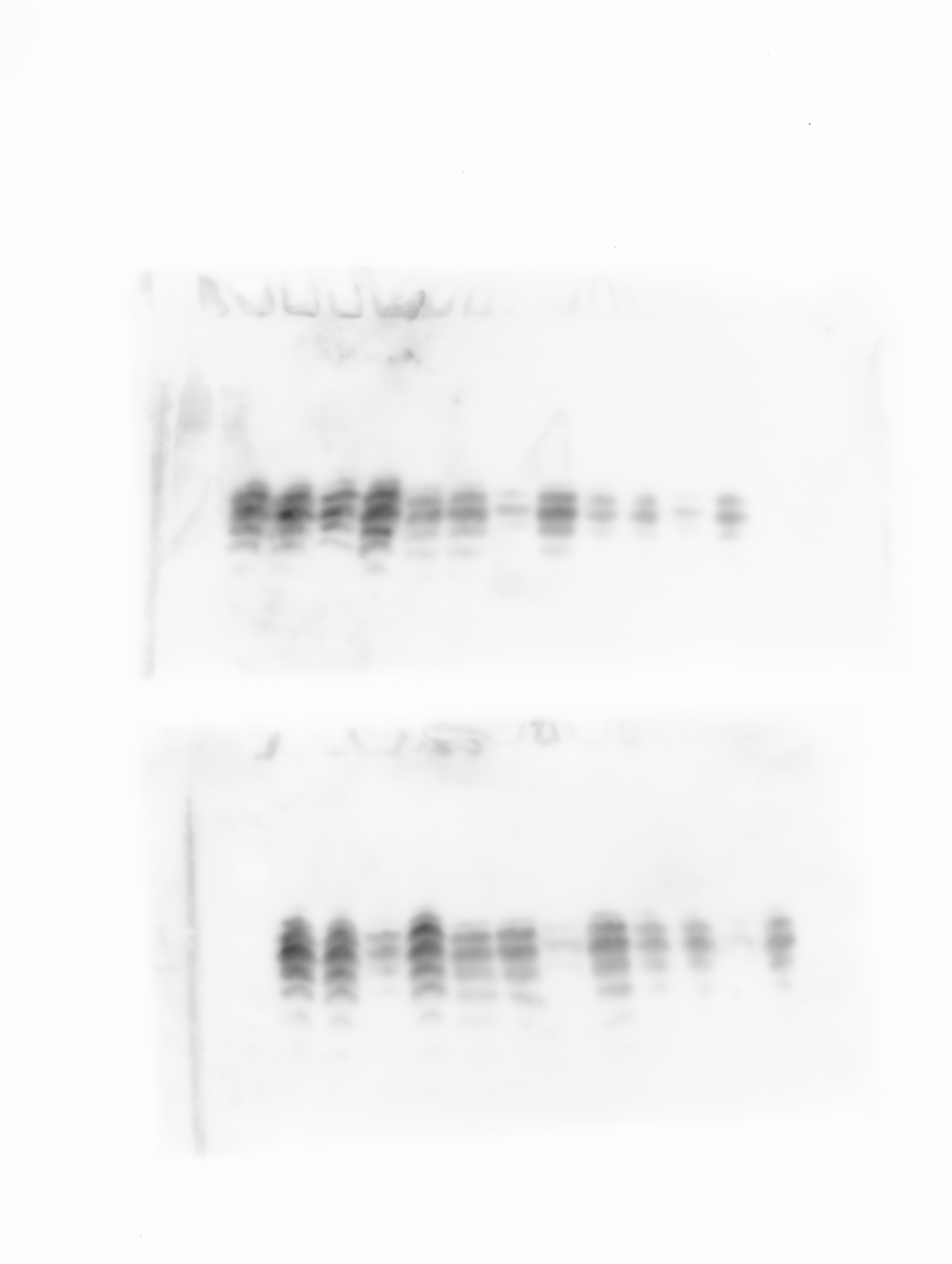

Supplement: Figure 8—figure supplement 1—source data 1. [file elife-76392-fig8-figsupp1-data1.zip › Figure 8 - figure suuplement 1 - source data/Figure 8 figure supplement 1 - source data_WTA_choline blot_.tiff]
