# Supplementary material for: WhyD tailors surface polymers to prevent premature bacteriolysis and direct cell elongation in Streptococcus pneumoniae
Source: eLife. 2022 May 20;11:e76392. doi: 10.7554/eLife.76392 (PMC9208761; doi:10.7554/eLife.76392)
Supplement: Supplementary file 1. — Transformations with indicated amplicons were performed as described in Materials and methods. Each transformation experiment was performed 3 times independently with similar results and the average number of colonies from the 3 experiments are shown. * Direct transformation of a ΔwhyD::spec amplicon into the indicated stains resulted in tiny colonies that were unable to grow in liquid media after 10 hr. [file elife-76392-supp1.docx]

| **Amplicon** | **Recipient strain** | **Number of colonies after 20 h after transformation** |
| --- | --- | --- |
| *ΔbgaA::spec* | WT (*D39 Δcps*) | >600 |
| *ΔwhyD::spec* |  | 5* |
| *ΔbgaA::spec* | *ΔlytA* | >450 |
| *ΔwhyD::spec* |  | 213 |
| *ΔbgaA::spec* | *ΔlytB* | >300 |
| *ΔwhyD::spec* |  | 12* |
| *ΔbgaA::spec* | *ΔlytC* | >400 |
| *ΔwhyD::spec* |  | 17* |
| *ΔbgaA::spec* | *ΔcbpD* | >200 |
| *ΔwhyD::spec* |  | 10* |
| *ΔbgaA::spec* | *ΔlytB ΔlytC ΔcbpD* | >200 |
| *ΔwhyD::spec* |  | 19* |

Supplementary File 1
