# Supplementary material for: WhyD tailors surface polymers to prevent premature bacteriolysis and direct cell elongation in Streptococcus pneumoniae
Source: eLife. 2022 May 20;11:e76392. doi: 10.7554/eLife.76392 (PMC9208761; doi:10.7554/eLife.76392)
Supplement: Supplementary file 2. [file elife-76392-supp2.docx]

**Supplementary File 2**

| **Name** | **Sequence** |
| --- | --- |
| AB_Marker_F | GAGGGAGGAAAGGCAGGA |
| AB_Marker_R | CGCCGTATCTGTGCTCTC |
| BgaA_5FLANK_F | CCTACATTTGATGACCTTCTTAACGCC |
| BgaA_5FLANK_R | AACTTCGTCAGTGTCGCCTTGC |
| *bgaA*_FLANK_F | GTTGCTACTAAACGTCTTCAAGGACG |
| *bgaA*_FLANK_R | CGCCAAACTTCAAGAACAGATACAGG |
| pLEM023_F | CACGATTACTTGGGGATCTCCCCGCGAAAGCGGG |
| pLEM023_R | TCCTGCCTTTCCTCCCTCGTCATACCATGTATACCACTTGG |
| *whyD*_5F_F | GCT CAAGCAGCGC ATACCCTCAA TCTCCCAGTC TTAGTCATCC |
| *whyD_*5F_R | TCCTGCCTTTCCTCCCTCAGGCACTCCTAGTCAAATAATTGAGACAAGTCCAAGCCACCAAAAGG |
| *whyD*_3F_F | GAGAGCACAGATACGGCGGCATTGGAATTCTTTGAATTTtagTAATAAAAAAGAGG |
| *whyD*_3F_R | CCAAGGCAGCCCAGCCTTCACAGACTCTACTGC |
| *whyD*_5F_SEQ | CGCAACAACCGCTTTATTTCGAATCAGACA |
| oSp95 | gccctcatcggaatacagac |
| oSp98 | ctgaagaatgtaaatggtgttaacttcgac |
| *lytA*_F_purification_NdeI | GCCCATATGGAAATTAATGTGAGTAAATTAAGAACAGATTTGCC |
| *lytA*_R_purification_HindIII | GCCAAGCTTttaTTTTACTGTAATCAAGCCATCTGGCTCTACTGTGAATTCTGG |
| *whyD*_CTERM_BAMHI_F_PTD68 | GGCGGATCCGTTC ATCTACCTCA ACTTATTTCT CACCGAGGGG TTTCCAATGC AAATGGGATT CAAAATACAG TAGAGTCC |
| *whyD*_CTERM_xhoI R_PTD68 | CCGCTCGAGctaAAATTCAAAGAATTCCAATGCTTTTTTCAAGAGCAAATCCGTATATTCTGGATCTTCTTGGGCTACTTCTATTTCCCG |
| *lytA*_F_nativeRBS_XhoI_F | TATCTCGAGTAATCGTGACTAAGATTGTCTTCTTTGTAAGG |
| *lytA*_F_BamHI_R | ataGGATCCCCTAATAATATGCGCTGTTCTGATTTGAAAGACATTCC |
| oGD267 | ggtgttcataaatctattataactcgagacaTAAGGAGGaactactatgagtaaaggagaagaac |
| oGD268 | tttgtatagttcatccatgccatgtg |
| oGD369 | cacatggcatggatgaactatacaaaCCTATGAAACCTGAAAAACCTAAAAAATTGGGTC |
| oGD270 | gcggggtttttttttggatccctaAAATTCAAAGAATTCCAATGC |
| oGD391 | GAATTCatAAGCTTgtCTCGAGtgctcgaagatttcagc |
| oGD392 (PF6- | gttcttctcctttactcattaattttcctccttatttatttagatcc |
| oGD67 | atgagtaaaggagaagaacttttcac |
| oGD193 | CCCGCTTCGGCGGGGTTTTTTTTTGGATCCttatttgtatagttcatccatg |
| *whyD*_optRBS_ATG_F_xhoI | TATCTCGAGacaTAAGGAGGaactactATGAAACCTG AAAAACCTAA AAAATTGGGT CTTAAACAGA TTTACCTCAA TCTTGATAAA ATCCTCTTTC |
| *whyD*_R_BamHI | *ataGGATCCCATTCACAAGAATACTTACCTATCATGGGAGGAACAACCG* |
| *lytB*_5F_F | GCCAACGCGTCTGTGAAGATGGTCC |
| *lytB _*5F_R | TCCTGCCTTTCCTCCCTCCCTCTGTTCTTATTTATTTTATTGTACC |
| *lytB* _3F_F | GAGAGCACAGATACGGCGGAGTGAATAGTAAGTTAAAAATCC |
| *lytB* _3F_R | CGATAAAAGCAGTCATAAAATCCTCG |
| *lytB* _5F_SEQ | GGGAAATGGTTTTGACTTGTCTGACC |
| *lytC*_5F_F | GAAGTACATTCAGGCGATTTTGAGAAACT |
| *lytC _*5F_R | TCCTGCCTTTCCTCCCTCGAATGCTGTTCCACCTAGCTTTTGC |
| *lytC* _3F_F | GAGAGCACAGATACGGCGCGCGTAGATGTCAGCGTTTGGTATTAA |
| *lytC* _3F_R | CAAACCAGGTGCTTGTCCAAGTTCG |
| *lytC* _5F_SEQ | CCAGTCCATTTCTAATCTGACGGAAAGG |

**SUPPLEMENTARY REFERENCES**

Fenton, A. K., Mortaji, L. E., Lau, D. T. C., Rudner, D. Z., & Bernhardt, T. G. (2016). CozE is a member of the MreCD complex that directs cell elongation in Streptococcus pneumoniae. *Nature Microbiology*, *2*(3), 16237. doi: 10.1038/nmicrobiol.2016.237

Flores-Kim, J., Dobihal, G. S., Fenton, A., Rudner, D. Z., & Bernhardt, T. G. (2019). A switch in surface polymer biogenesis triggers growth-phase-dependent and antibiotic-induced bacteriolysis. *ELife*, *8*, e44912. doi: 10.7554/elife.44912

Lanie, J. A., Ng, W.-L., Kazmierczak, K. M., Andrzejewski, T. M., Davidsen, T. M., Wayne, K. J., … Winkler, M. E. (2007). Genome Sequence of Avery’s Virulent Serotype 2 Strain D39 of Streptococcus pneumoniae and Comparison with That of Unencapsulated Laboratory Strain R6▿ ‡. *Journal of Bacteriology*, *189*(1), 38–51. doi: 10.1128/jb.01148-06

Liu, X., Gallay, C., Kjos, M., Domenech, A., Slager, J., Kessel, S. P., … Veening, J. (2017). High‐throughput CRISPRi phenotyping identifies new essential genes in Streptococcus pneumoniae. *Molecular Systems Biology*, *13*(5), 931. doi: 10.15252/msb.20167449

Uehara, T., Parzych, K. R., Dinh, T., & Bernhardt, T. G. (2010). Daughter cell separation is controlled by cytokinetic ring‐activated cell wall hydrolysis. *The EMBO Journal*, *29*(8), 1412–1422. doi: 10.1038/emboj.2010.36

Wang, X., Tang, O. W., Riley, E. P., & Rudner, D. Z. (2014). The SMC Condensin Complex Is Required for Origin Segregation in Bacillus subtilis. *Current Biology*, *24*(3), 287–292. doi: 10.1016/j.cub.2013.11.050

Youngman, P. J., Perkins, J. B., & Losick, R. (1983). Genetic transposition and insertional mutagenesis in Bacillus subtilis with Streptococcus faecalis transposon Tn917. *Proceedings of the National Academy of Sciences*, *80*(8), 2305–2309. doi: 10.1073/pnas.80.8.2305
